# Student and staff experiences of interactive digital models when learning animal anatomy

**DOI:** 10.1002/ase.70286

**Published:** 2026-06-11

**Authors:** Alice Leavey, Sarah Channon

**Affiliations:** ^1^ Department of Cell and Developmental Biology, Centre for Integrative Anatomy UCL London UK; ^2^ Comparative Biomedical Sciences Royal Veterinary College London UK

**Keywords:** digital anatomy, pedagogy, veterinary education, virtual dissection

## Abstract

Compared to human medical sciences, digital models of animals are rare, often behind subscription services and are comparatively lacking in interactivity and accessibility features. This study explores how digital models are currently being used to teach animal anatomy, providing the data required to construct the best anatomical models possible, to best suit the needs of a diverse student community. We designed an online survey which was completed by 35 students and eight staff learning and teaching veterinary and biological sciences from six universities across the United Kingdom. The results showed students struggle most with learning topographically vast body regions, such as the nervous and endocrine systems, and the anatomy of exotic and equine species. We find that digital models are being used more frequently by those on veterinary degrees compared to the biological sciences, often to consolidate learning after anatomy practicals. However, most participants were not aware of the existing resources we asked them to rate. Participants agreed that better digital models are needed in anatomy education, which would help students prepare for their studies and future careers. The most important features of the ideal digital anatomical model were being free to use and easy to navigate, with highly detailed annotations which can be toggled on and off to test anatomical knowledge. Participants agreed that while implementation of this ideal digital model would facilitate learning, virtual dissection practicals should not replace traditional anatomy practicals due to the valuable skills gained from hands‐on experience with real tissue and dissection tools.

## INTRODUCTION

Digital anatomical models (DAMs) allow students to manipulate 3D reconstructions of anatomy, explore structures in detail, and apply their theoretical knowledge of complex medical concepts. Students can rotate, zoom, and dissect models at their own pace, making the learning experience more personalized and improving spatial comprehension, which is vital for understanding the intricacies of anatomy.^1^, ^2^ DAMs provide the means to perform non‐invasive dissections of virtual bodies, facilitating greater access to students at institutions with fewer resources for performing traditional laboratory dissections, as they can be readily accessed via computers.^3^ DAMs provide a repeatable and standardized experience that can enable students to develop decision‐making skills in a low‐risk environment.^4^ Additionally, provided that consent has clearly been given by the donor of the medical images, DAMs also raise fewer ethical concerns.^5^, ^6^ Using DAMs in conjunction with augmented/virtual reality and 3D printing technologies also creates immersive and tactile learning experiences.^7^, ^8^, ^9^, ^10^, ^11^, ^12^, ^13^

In human medical education, DAMs and similarly advanced teaching technologies are increasingly integrated into curricula to enhance understanding and support interactive learning of anatomy.^14^, ^15^ There are many websites, apps, and companies entirely dedicated to visualizing human DAMs (e.g., Complete Anatomy, Anatomy 3D Atlas, Anatomage). Demand for such tools in medical education has grown in recent years due to the increase in the number of new medical schools now offering courses that avoid body donor use entirely (e.g., University of Lancashire, University of Oxford^16^). In comparison, high‐quality DAMs of animals are sparse,^17^ with comprehensive atlases of the entire animal body are even rarer.

Veterinary sciences require in‐depth knowledge of the anatomy of multiple species. While the COVID‐19 pandemic saw a notable shift from dissection‐based learning to digital platforms,^3^, ^18^ veterinary anatomy educators have less access to high‐quality digital resources. Many such resources are stored in private university libraries or, as with several models in human medical education, behind subscription paywalls. Some of these are completely inaccessible to students studying biosciences curricula, despite the fundamental relevance of animal physiology and comparative anatomy in facilitating understanding of the normal animal and diversity of life. Overall, this places significant limitations on the ways that vertebrate anatomy, development, function, and evolution are taught in both schools and higher education institutions.

This exploratory study addresses the limited evidence on how DAMs are currently used to facilitate learning in the anatomical sciences within biosciences and veterinary courses across UK universities. We aim to identify the anatomical areas in which students and staff require the most support to gain insight into how DAMs could be best utilized to enhance student performance and learning satisfaction. We also quantify which features of DAMs students and staff perceive to be most important in order to enable future educators and researchers to create high‐quality educational resources.

## METHODS

Ethical approval for this study was obtained through UCL's central Research Ethics Service (Ethics ID 28615/001), and the study format was designed following the recommendations of Wilson et al.^19^

### Survey distribution and incentivization

The target survey population consisted of students and teaching staff on undergraduate or postgraduate courses related to anatomical sciences at UK universities (e.g., biology, zoology, veterinary medicine). All participants were above the age of 18 and resided primarily in the United Kingdom. To be eligible to complete the survey, students were required to be currently studying in their course or have graduated since 2021, in order to avoid biases through pre‐COVID‐19 experiences, when the implementation or use of digital models may not have been planned or optimal. Participants were required to have completed at least one animal anatomy practical (e.g., dissection lab, museum visit, handling of fixed/skeletonized specimens) to be eligible. For staff to be eligible, they had to be currently involved with teaching at least one anatomy practical session.

Ensuring participant eligibility was primarily controlled by means of distribution. The survey was shared via email to module leaders across eight of the 10 UK universities that offer relevant degree courses. Module leaders were asked to then share the survey with their students. This multi‐time point survey was hosted on Microsoft Forms and released for 6 weeks toward the end of each 2024/2025 academic term (November/December 2024 and March/April 2025). This avoided exam periods while also providing enough time for students who are likely to have completed an anatomy practical recently. During the second distribution window, the survey was distributed throughout a wider network of colleagues and universities in order to support an increase in survey responses. To ensure that the target audience completed the survey only once, email addresses were checked for repeats and responses were examined to ensure none were identical. Two email reminders were sent—one a week before the survey deadline and one the day before. At University College London (UCL) and the Royal Veterinary College (RVC), the survey was also disseminated via one staff bulletin and in the form of posters inside buildings around campus. Participant eligibility was also verified through survey questions, which requested information on the degree course and year of study currently being completed (student) or most frequently taught on (staff).

The survey was voluntary. Survey incentives were offered in the form of a prize draw determined by a random number generator corresponding to participant number. To participate in the prize draw, participants were instructed to provide their academic email address—another form of verification for survey eligibility.

### Survey design

Prior to starting the survey, participants were provided with a glossary of terms to avoid ambiguity. “Anatomy practicals,” primarily referred to the physical dissection, manipulation and/or handling of specimens, whether fresh, fixed (i.e., preserved in formalin), plastinated, skeletonized, fossilized, or as 3D printed replicas. This can include practicals where specimens are handled by only staff, including postgraduate teaching assistants, where students simply observe and take notes. We also clarified that anatomy practicals can take place in multiple settings (e.g., lab, museum, zoo). “Physical anatomical models” referred to fixed and skeletonized specimens (Figure 1A), and 3D prints (Figure 1B), which can usually be found around buildings teaching biological and veterinary sciences. “Digital anatomical models” refer to interactive 3D structures that can be viewed and manipulated on websites or apps (e.g., Sketchfab.com; Figure 1C). Digital models do not include static 2D images on a PowerPoint, for instance. They usually have accompanying annotations and sometimes animations.

**FIGURE 1:**
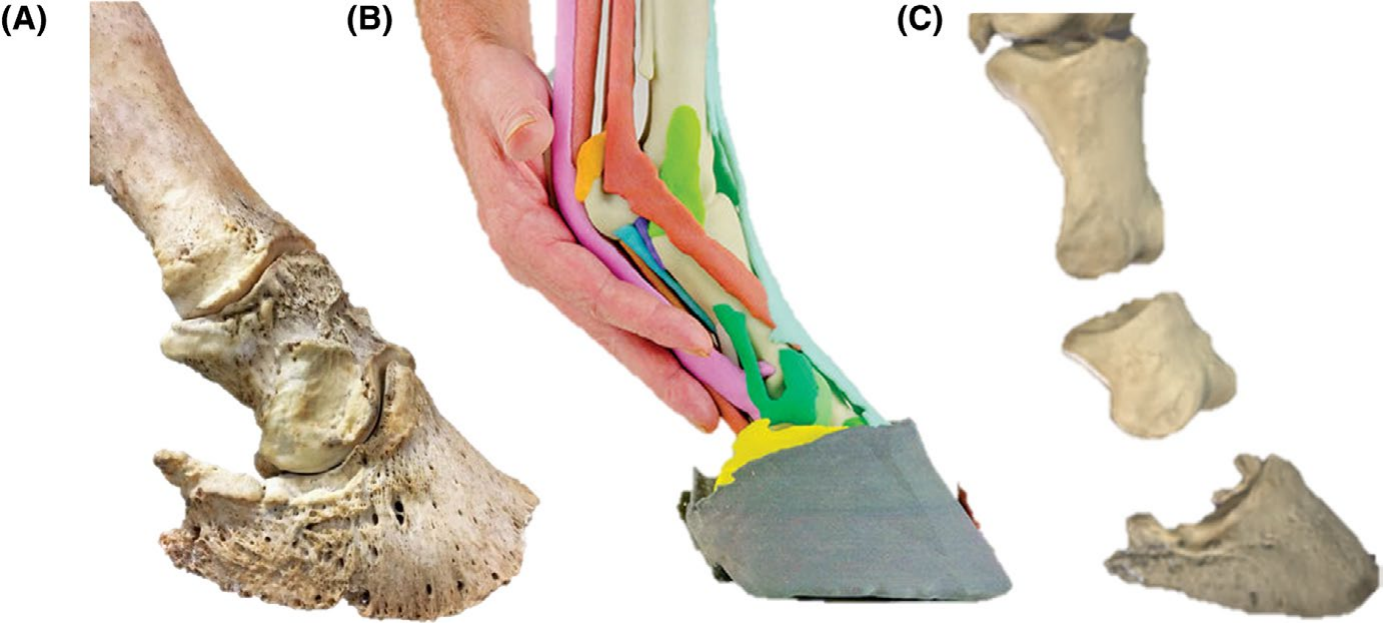
Example of the different types of anatomical models commonly used in practicals, demonstrated by the distal limb of a horse. (A) A physical anatomical model in the form of a real, skeletonised specimen (image credit: *Walter Varcoe*, equineskeletons.com); (B) *a physical anatomical model* in the form of a 3D‐printed model (image credit: *3D VetAnatomize*); (C) an interactive digital anatomical model created from a surface scan of a real skeleton (credit: *University of Dundee Museum Collections;* sketchfab.com).

This survey was designed to improve our understanding of what students are required to do when learning animal anatomy, how interactive digital resources currently facilitate learning in animal anatomy, and what the most important criteria are for creating the most useful digital anatomical models possible. To reflect this, participants were asked to respond to the following sections:

- Section 1: About you.
- Section 2: Your experience learning/teaching anatomy so far.
- Section 3: Your anatomy practicals.
- Section 4: Your use of anatomical models so far.
- Section 5: What would the ideal digital anatomical model be?
- Section 6: How digital anatomical models impact student learning.

All participants were asked two yes/no questions at the beginning of the survey to ensure they agreed to the data protection privacy information provided and to obtain their consent to participate and use their answers for future research. We highlighted the option to withdraw at any time before submission. The main section of the survey consisted of 34 key questions: 4 single‐choice categorical questions; 8 multiple‐choice categorical questions; 10 Likert scale questions, which were further subdivided into 59 survey items; and 11 free‐response text questions designed to collect more detailed reasoning for responses, 10 of which were optional (Appendices A and B). Participants were then asked two yes/no questions asking whether participants wanted to opt into the prize draw and a potential future focus group. The final, optional text question was to provide their academic email address for these purposes.

### Pilot test

Prior to full‐scale distribution, the first draft of the survey was sent out to four volunteers from UCL and RVC with expertise in anatomy and/or experience performing survey‐based studies. These participants were requested to complete a feedback form designed to evaluate the content validity and reliability of each survey section, in line with the Standards for Educational and Psychological Testing (see Supporting Information). The main feedback from this pilot test was that wording the questions to try to be relevant to both students and teaching staff simultaneously was potentially confusing. Therefore, the final version of the survey was delivered in two similar formats—one phrased with students in mind, and the other phrased for teaching staff (see Supporting Information for full versions of both surveys). All members of this pilot group confirmed that the final version of the survey integrated their feedback prior to full‐scale distribution, and only their response to the final version was included in the statistical analysis.

### Statistical analysis

All analyses were performed in R (version 2022.07.2). The data were checked manually for signs of repeated attempts and improper completion (i.e., very short survey times, same answers for every question), and none were deemed necessary to remove. Responses from the student and staff surveys were analyzed both separately and pooled together. A series of independent variables were analyzed in this study, mostly related to survey demographics—whether the participant is staff or student, their primary role (e.g., undergraduate, teaching staff), their primary degree course (e.g., veterinary medicine, biology), their previous experience with animal anatomy, and the frequency of anatomy practicals per year.

The total number of Likert scale questions (*n* = 59) was separated into themes that reflected Sections 2–6 of the survey: general experiences with anatomy, information on practicals, opinions on practicals, the ideal DAM, and impacts of DAMs. These themes were tested for internal consistency (i.e., how closely related each set of items is). First, the Likert questions relating to the rating of existing DAMs were removed from further analysis due to the high number of “NA” responses. Second, responses were numerically coded for analysis, where positive responses were coded to be larger (i.e., strongly disagree = 1, strongly agree = 5). Negatively phrased Likert statements were reverse‐coded to ensure that all were measured on a scale with the same directionality. Cronbach's alpha was calculated for the overall reliability across all analyzed Likert questions and within each theme. Using the original data, Kendall's tau‐b was calculated among all pairs of Likert questions, where larger values suggest strong associations between the questions. Each Likert scale question was then analyzed for significant differences between independent variables using Kruskal–Wallis tests. A post hoc Dunn's test was performed where the overall difference between groups was significant to examine pairwise relationships. Responses to the six multiple‐choice questions were analyzed for differences between independent variables using a chi‐squared test, where residuals were examined to determine the highest contributing combinations.

For the 11 free‐text responses, a summative content analysis was performed manually by a single researcher, where the most common words or phrases that occurred were recorded. For example, we report the frequency each animal was mentioned in response to questions 10 (*For which animals do you find the anatomy most difficult to learn/teach, and why?*) and 30 (*Which animal(s) would you benefit from most by having a digital anatomical model?*). For relevant questions, this was followed by a contextual examination of the terms (i.e., whether they were positive or negative, framed theoretically or factually). Representative quotes were also selected to illustrate responses to each theme in the survey (see above), ensuring diversity of perspectives (i.e., from both staff and students, from different degree subjects) and inclusion of both typical and divergent views.

## RESULTS

Eight academic staff and 35 students across six universities responded to the survey. We cannot accurately calculate the absolute response rate, as the distribution was reliant on sharing our survey with a large network of colleagues, who then shared the survey with their students, making it difficult to know the total number of invited participants. The staff survey was primarily completed by those who teach first and second‐year students studying veterinary medicine (Table 1). Most of the student survey respondents were first and second‐year undergraduates studying biology or veterinary medicine, and they had previous experience with anatomy from before their current degree (Table 2). Additionally, out of the participants who chose to volunteer the information (*n* = 33), several reported that they are neurodivergent (18%), have a mental health condition (23%) or a form of disability (5%).

**TABLE 1:** Staff survey demographics (total respondents = 8).

Variable	Values	Number	Percentage
Primary occupation	Teaching staff	5	63%
	Research staff	2	25%
	Other—Technical staff	1	12%
Most commonly taught year group(s)	First‐year undergraduate	7	88%
	Second‐year undergraduate	6	75%
	Third‐year undergraduate	2	25%
	Fourth‐year undergraduate	2	25%
	Other— “Pre‐university, Graduate Entry”	1	13%
Most commonly taught degree course	Veterinary medicine	6	75%
	Biology	2	25%
Previous anatomy experience(s)	School	3	38%
	Undergraduate degree	7	88%
	Postgraduate degree	4	50%

**TABLE 2:** Student survey demographics (total respondents = 35).

Variable	Values	Number	Percentage
Student type	Undergraduate—first degree	29	83%
	Undergraduate—second degree	3	9%
	Postgraduate student	1	2%
	Recent graduate not currently in education	2	6%
Year of current course	First	15	43%
	Second	16	46%
	Third	1	3%
	Fourth	1	3%
	NA (recent graduate)	2	5%
Degree Course	Veterinary Medicine	13	37%
	Veterinary Nursing	4	11%
	Biology	15	43%
	Zoology	3	9%
Previous anatomy experience(s)	No	9	26%
	Pre‐university	23	66%
	Undergraduate degree	5	14%

The responses to the Likert scale questions were deemed reliable for further interpretation (Cronbach's alpha = 0.832). The statements with the weakest reliability score were those within the theme of “the ideal DAM,” likely because there are only three statements in this section (Table S1). “Learning anatomy” also showed a moderate score, which improved marginally with the removal of statements with some NA responses (Table S1). The remaining statements all showed a score higher than 0.7, the current consensus for the desirable level of survey consistency (Taber^20^).

### Common experiences when learning and teaching anatomy

All but one survey participant agreed that anatomy is an enjoyable subject to learn or teach (Figure 2). Participants who agreed more strongly were significantly associated with the opinion that they tended to score well on topics related to anatomy (Kendall's tau‐b: −0.366, *p* = 0.012; Figure S1). In general, students tend to score well on anatomy‐focused assessments regardless of degree type (Table 3). Most participants agreed that they find it easy to understand how one small anatomical part fits into a wider system, while a narrow majority agreed they find it easy to understand how structures move in 3D space (Figure 2). Those who agreed with the former statement were significantly more associated with agreeing to the latter (Kendall's tau‐b: 0.461, *p* < 0.001; Figure S1). Those who found it easier to understand movement in 3D space also said that they scored well on anatomy‐based assessments significantly more often (Kendall's tau‐b: −0.334, *p* = 0.014; Figure S1).

**FIGURE 2:**
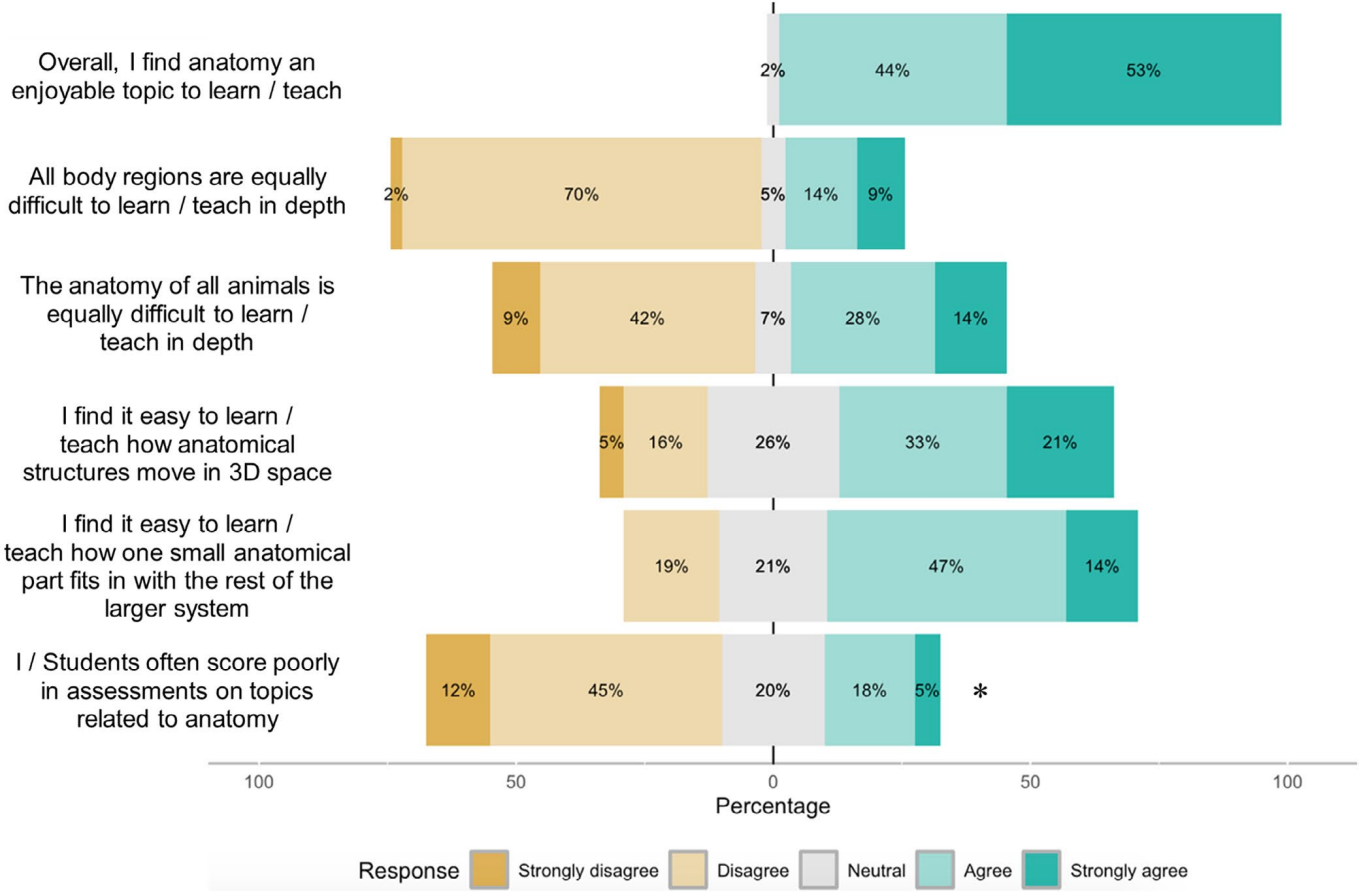
Combined survey responses to statements themed around learning (student survey) and teaching (staff survey) anatomy, on a scale from strongly disagree to strongly agree, are reported in the order they appear on the survey. The asterisk indicates a significant difference in how undergraduates responded: Those on their second degree agreed more strongly that they score poorly on anatomy assignments than recent graduates (*p* < 0.05; Table 3).

**TABLE 3:** The results of Kruskal–Wallis (KW) and Dunn's tests for each Likert scale question that showed significant differences between independent variables after *p*‐value adjustment with Bonferroni correction for multiple testing (*p < 0.05; **p < 0.01; ***p < 0.001).

Dataset	Statement	Independent variable	KW chi‐squared	KW *p*‐value	Comparison	Effect size	Unadjusted *p*‐value	Adjusted *p*‐value
Staff survey responses	Students watch a demonstrator handle a fresh/fixed specimen (rated “never” to “always” done in a practical)	Subject	5.333	0.021*	Biology‐Veterinary Medicine	2.309	0.010	0.010*
	Students watch a demonstrator handle a skeleton/fossil (rated “never” to “always” done in a practical)	Subject	4.48	0.034*	Biology‐Veterinary Medicine	2.117	0.017	0.017*
	The skills that students learn in anatomy practicals will remain relevant as they go on to the next stage of their career	Subject	6.857	0.009**	Veterinary Medicine‐Biology	2.619	0.004	0.004**
	Being provided with good digital anatomical models would help students to rely less on their peers	Subject	4.481	0.034*	Biology‐Veterinary Medicine	2.117	0.017	0.017*
Student survey responses	I often score poorly in assessments on topics related to anatomy	Participant type	8.146	0.043*	Undergraduate (second degree)‐Recent graduate	2.642	0.004	0.025*
	Skeletal anatomy (rated from “very challenging” to “very easy”)	Experience	9.259	0.010*	Undergraduate‐No	3.035	0.001	0.004**
					Undergraduate‐School	2.132	0.016	0.049*
	Digestive anatomy (rated from “very challenging” to “very easy”)	Year group	9.535	0.049*	First‐Second	2.634	0.004	0.042*
	Cardiovascular anatomy (rated from “very challenging” to “very easy”)	Participant type	10.472	0.015*	Recent graduate‐Undergraduate (second degree)	2.818	0.002	0.015*
					Undergraduate (first degree)‐Undergraduate (second degree)	2.488	0.006	0.039*
	Respiratory anatomy (rated from “very challenging” to “very easy”)	Experience	7.936	0.019*	Undergraduate‐No	2.788	0.003	0.008**
	Reproductive anatomy (rated from “very challenging” to “very easy”)	Year group	13.309	0.010*	First‐Second	2.947	0.002	0.016*
	Physically dissect a fresh/fixed specimen (rated “never” to “always” done in a practical)	Year group	18.987	<0.001***	Graduate‐First	3.032	0.001	0.012*
					Second‐First	3.638	0.000	0.001**
	Physically handle a fresh/fixed specimen (rated “never” to “always” done in a practical)	Subject	17.191	<0.001***	Veterinary Medicine‐Biology	3.878	0.0001	<0.001***
					Veterinary Medicine‐Veterinary Nursing	2.494	0.006	0.038*
	Physically handle a fresh/fixed specimen (rated “never” to “always” done in a practical)	Practical frequency	14.192	0.014*	Weekly‐Twice a year	3.285	0.001	0.008**
	Physically handle a skeleton/fossil (rated “never” to “always” done in a practical)	Subject	11.845	0.008**	Veterinary Medicine‐Biology	3.106	0.001	0.006**
	Physically handle a skeleton/fossil (rated “never” to “always” done in a practical)	Practical frequency	11.865	0.037*	Weekly‐Twice a year	2.783	0.003	0.040*
	Watch a demonstrator handle a skeleton/fossil (rated “never” to “always” done in a practical)	Year group	9.501	0.050*	First‐Second	2.622	0.004	0.044*
	My anatomy practicals so far have been safe	Year group	12.249	0.016*	First‐Second	2.725	0.003	0.032*
	The skills learned in anatomy practicals will remain relevant as I go on to the next stage of my career	Subject	10.508	0.015*	Veterinary Medicine‐Biology	2.652	0.004	0.024*
					Veterinary Medicine‐Zoology	2.453	0.007	0.043*
	I would prefer having access to models of an entire juvenile specimen over a model of only one part of an adult specimen	Year group	11.481	0.022*	First‐Second	2.596	0.005	0.047*
Comparing student and staff responses	Physically handle a fresh/fixed specimen (rated “never” to “always” done in a practical)	Group	4.475	0.034*	Staff‐Student	2.115	0.017	0.017*
	Watch a demonstrator handle a fresh/fixed specimen (rated “never” to “always” done in a practical)	Group	3.975	0.046*	Staff‐Student	1.994	0.023	0.023*
	Being provided with good digital anatomical models would help me/students to rely less on my/their peers	Group	7.843	0.005**	Student‐Staff	2.801	0.003	0.003**

*Note*: The effect sizes have all been presented as positive (i.e., the first variable in the comparison is always larger/more positive). The results are presented in the same order as presented in the survey.

The majority of participants reported that each region of the body varies in how difficult it is to learn/teach (Figure 2). A more detailed investigation revealed that the nervous, muscular, endocrine, and lymphatic systems are the most challenging (Figure 3). Participants with previous undergraduate experience with anatomy (i.e., as a staff member, postgraduate, or someone on their second undergraduate degree) found skeletal and respiratory anatomy easier than those with no experience (Dunn's test: 3.04; *p* < 0.01), or experience from school (Dunn's test: 2.13, *p* < 0.05; Table 3). Digestive and reproductive anatomy was also significantly easier for first‐year undergraduates compared to second years (Dunn's test: 2.95, *p* < 0.01; Table 3). There were no significant differences between degree subjects in which anatomical areas participants found challenging. Free‐text comments gave some further depth of understanding into which systems are challenging to learn/teach and why this might be:

I particularly struggle with the anatomy of the **nervous system** because it is so vast. **3D models do make it slightly easier** ‐ they give me an understanding on how the system works as a whole and better interlinked with other systems. [Student, veterinary medicine]

I find the **endocrine system** very tricky to learn as I **cannot look at something physical** like a skeleton. [Student, biology]

When we have to write an SAQ about origins, insertions, blood supply, and innervation, I find that its **a lot to remember for each and every muscle** and it lacks context really, I think it would be better maybe if we could look at the muscle, see the nerves that innervate it and the blood vessels suppling it, in a 3D way rather than in words. [student, veterinary medicine]

Students seem to struggle a lot with **terminology for movements** (e.g., abduction‐adduction) and **planes of direction**. [Staff, veterinary medicine]

The most challenging areas to teach are ones where the relevant structures are either **difficult to see or encased in a hard structure**… or where the relevant structures are **many, and separate, distributed widely** around the body ‐ this requires students to either link together knowledge from separate specimens/sessions, or for us to use an entire cadaver to demonstrate that system, which is not necessarily good use of a whole cadaver. [Staff, veterinary medicine]

When asked about learning/teaching anatomy for different animals, 51% of participants reported that they find the level of difficulty varies (Figure 2). This answer was strongly associated with agreeing to the statement that body regions vary in difficulty (Kendall's Tau‐b: 0.507, *p* < 0.001; Figure S1). Exotics (i.e., reptiles, fish, amphibians) were mentioned most often as being the most difficult (Figure S3), largely because less is known about them, and they are difficult to acquire for dissection practicals. Equine was the second‐most difficult non‐human animal—participants reported that this was largely because of the space and logistics associated with teaching using live or whole animal bodies, as well as how much their anatomy differs from other mammals.

**FIGURE 3:**
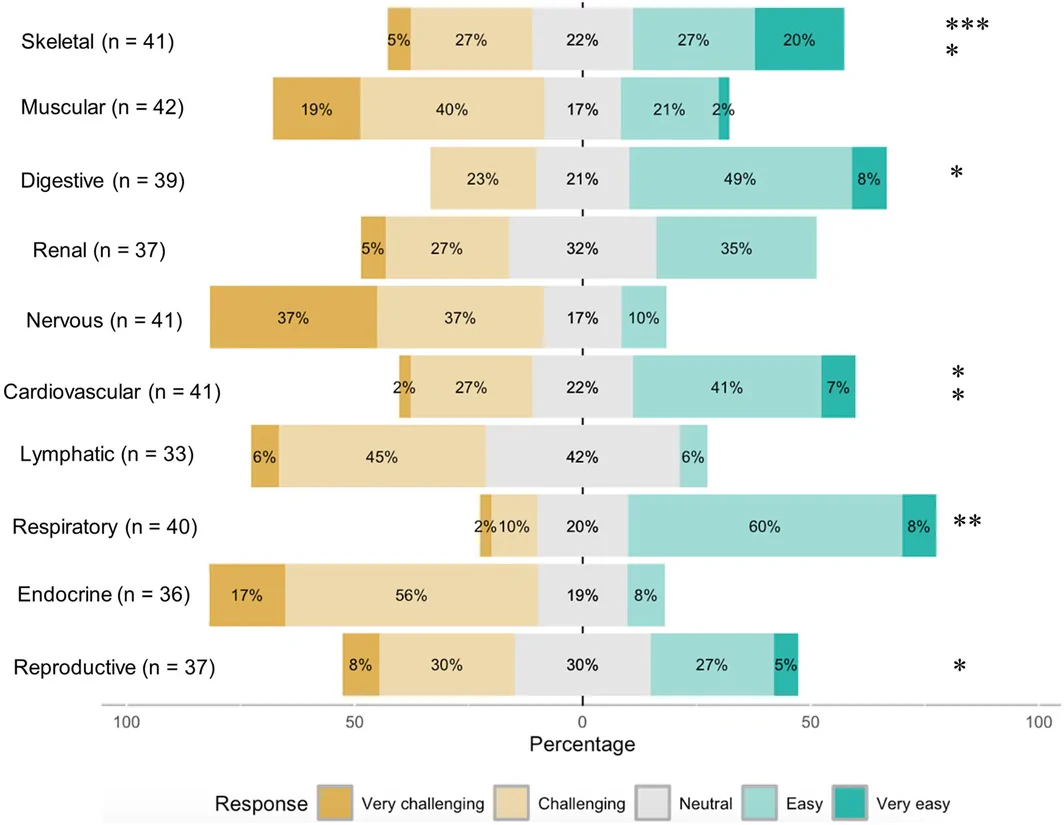
Combined survey responses to how difficult each anatomical system is to learn (student survey)/teach (staff survey), on a scale from very challenging to very easy, reported in the order they appear on the survey. As some responses were “NA,” response numbers have been reported for each anatomical system (total survey *n* = 43). The asterisks indicate that there are several significant differences between independent variables for responses from students (*p < 0.05; **p < 0.01; ***p < 0.001; summarized in Table 3).

### Anatomy practicals

There is a significant difference between degree subjects in how frequently animal anatomy practicals take place (chi‐squared: 43.41, *p* < 0.001), where veterinary medicine students have them more often (Figure 4). The practicals include a broad range of activities (Figure 5). In general, fresh/fixed specimen dissection happens less often than the simple handling of them, but the activity primarily depends on the degree course, frequency of practicals in a given year, and the year of study (Table 3). To summarize, students in veterinary medicine handle fresh/fixed and skeletal specimens significantly more often than those studying biology, who would most often watch demonstrations of these activities by staff. Handling of specimens also occurs significantly more for students who had anatomy practicals on a weekly basis compared to those who have them just twice a year (Table 3). Additionally, second‐year students and recent graduates physically dissect specimens significantly more than first‐year students, who primarily watch staff handle skeletal specimens (Table 3).

**FIGURE 4:**
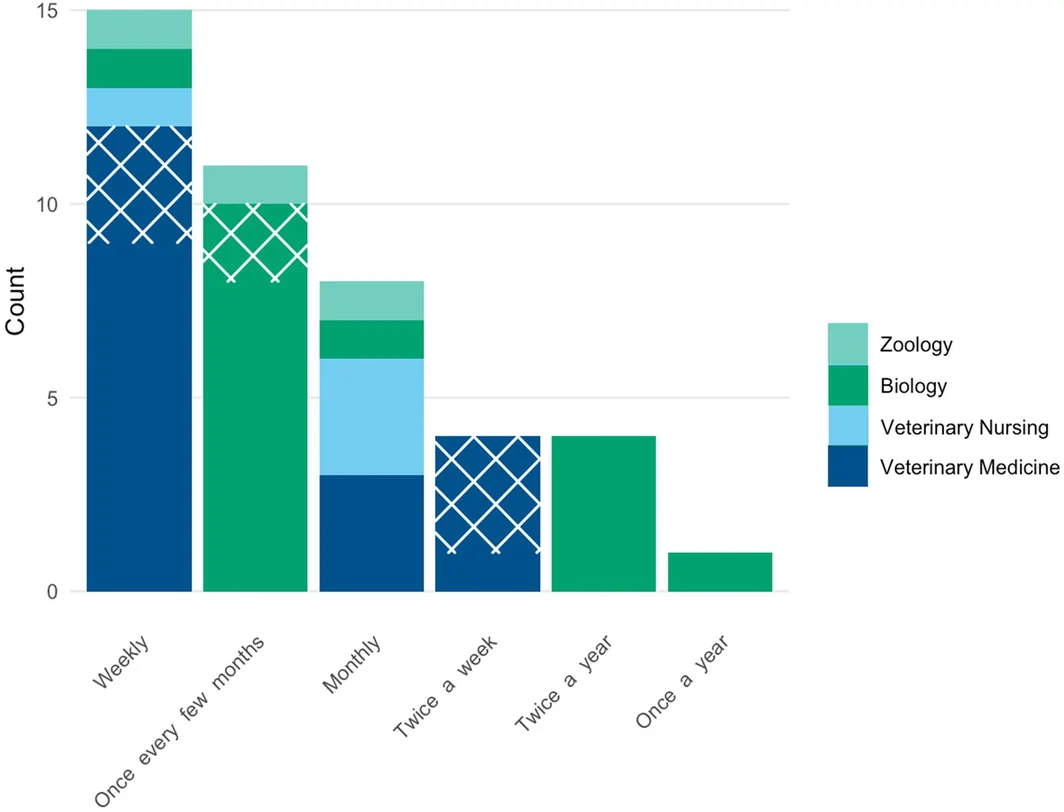
A stacked bar plot presenting the frequency of student anatomy practicals in a given year, color‐coded by participant degree subject. Crosshatch patterns represent the responses from staff.

**FIGURE 5:**
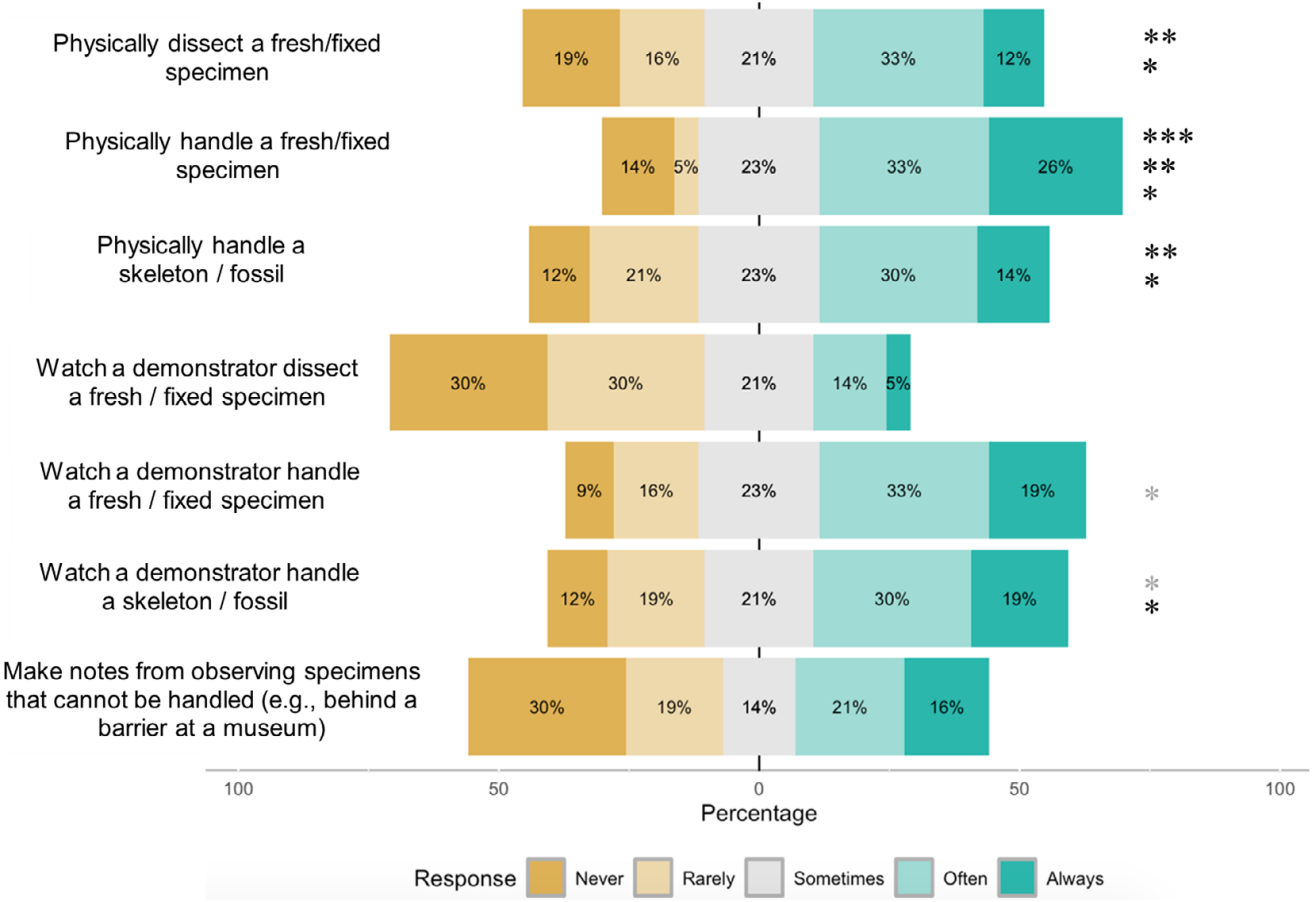
Combined survey responses to statements themed around what is involved in anatomy practicals, on a scale from never to always. The asterisks indicate that there are several significant differences between independent variables examined in this study, (*p < 0.05; **p < 0.01; ***p < 0.001; summarized in Table 3). The asterisks in black refer to results from the student survey, while the asterisks in gray refer to results from the staff survey.

The types of activities students are required to do in advance of anatomy practicals, and what activities they are required to do afterwards to consolidate their knowledge, were also recorded (Figure 6). The review of lecture content was consistent both before and after, while video demonstrations and directed reading were favored more as the method of practical preparation, with quizzes being the primary method of consolidation. Significant differences were found between post‐practical activities according to participant type (chi‐squared: 61.36, *p* < 0.001) and year group (chi‐squared: 60.23, *p* = 0.007), where students in the later years of their academic journey (fourth‐year students, recent graduates, and postgraduates) were more often required to complete presentations and written assignments (Table S2).

**FIGURE 6:**
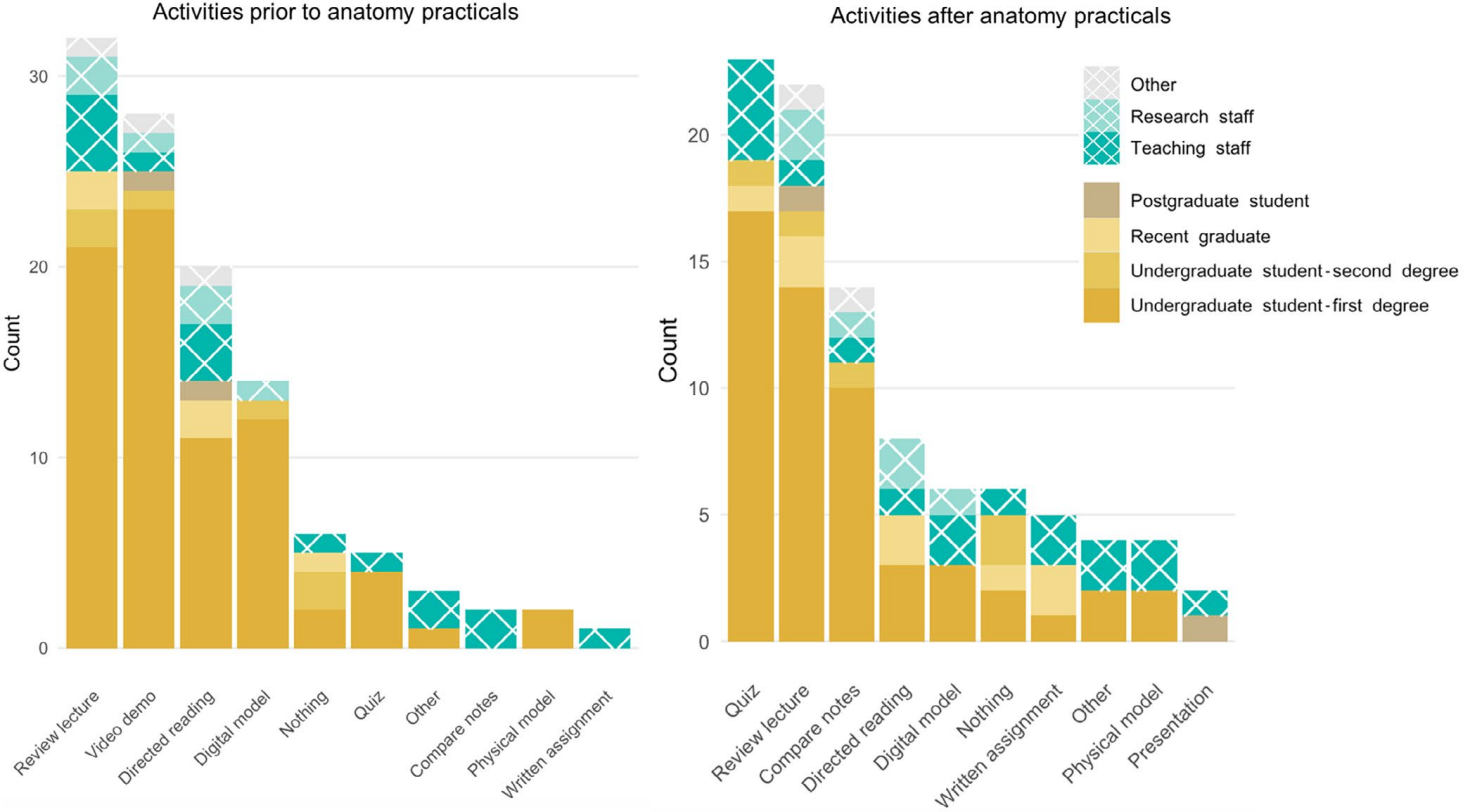
Stacked bar plots displaying responses to the multiple‐choice question on what activities students are required to do in advance to prepare for anatomy practicals, and what activities they are required to do afterwards to consolidate learning, color‐coded by survey participant type. Crosshatch patterns represent the responses from staff.

Regarding the qualities of the anatomy practicals, the responses were very positive, where the most disagreement was found regarding the ethical (7%, of which 2% was strongly disagree), interactive (7%), appropriately challenging (7%), and engaging (5%) aspects (Figure S2). When asked what other skills, capabilities, or awarenesses these practicals help students to develop, the most frequently mentioned themes were tissue handling (*n* = 5), understanding the body as a whole (i.e., the context of size and location of organs in 3D space) (*n* = 4), manual dexterity (*n* = 3), teamwork (*n* = 3), and confidence (*n* = 3).

Fifty‐nine per cent of survey participants agreed that their current curriculum (i.e., lectures and online learning material, such as pre‐practical activities) provides sufficient information for students to feel prepared to enter their anatomy practicals (Figure 7). While the majority agreed that the current range of anatomy practicals they are provided is diverse and interesting, many wish that there were more scheduled earlier (65%) and later (56%) in their undergraduate course (Figure 7). Participants studying veterinary medicine agreed significantly more often that the skills learned during anatomy practicals will remain relevant in the next stage of their careers than those studying biology and zoology, in both the staff and student surveys (Table 3). When asked how participants believed their anatomy practicals could be improved, five participants mentioned a need for more dissections, four mentioned the need for a longer time for preparation and three mentioned a need for further explanation of the practical's content:

Space and time does not allow for it, but longer on some practicals, and **more specimens to dissect, in smaller groups**. [Staff, veterinary medicine]

Incorporating 3D models [into anatomy practicals would better prepare students for their studies], but are **often not available for many taxa**. [Staff, biology]

Making sure we have a full understanding of any content before the practical and not introducing new material in the lab. [Student, biology]

Sometimes the **practical sheets use very complex language** making the actual steps difficult to understand. [Student, veterinary medicine]

**FIGURE 7:**
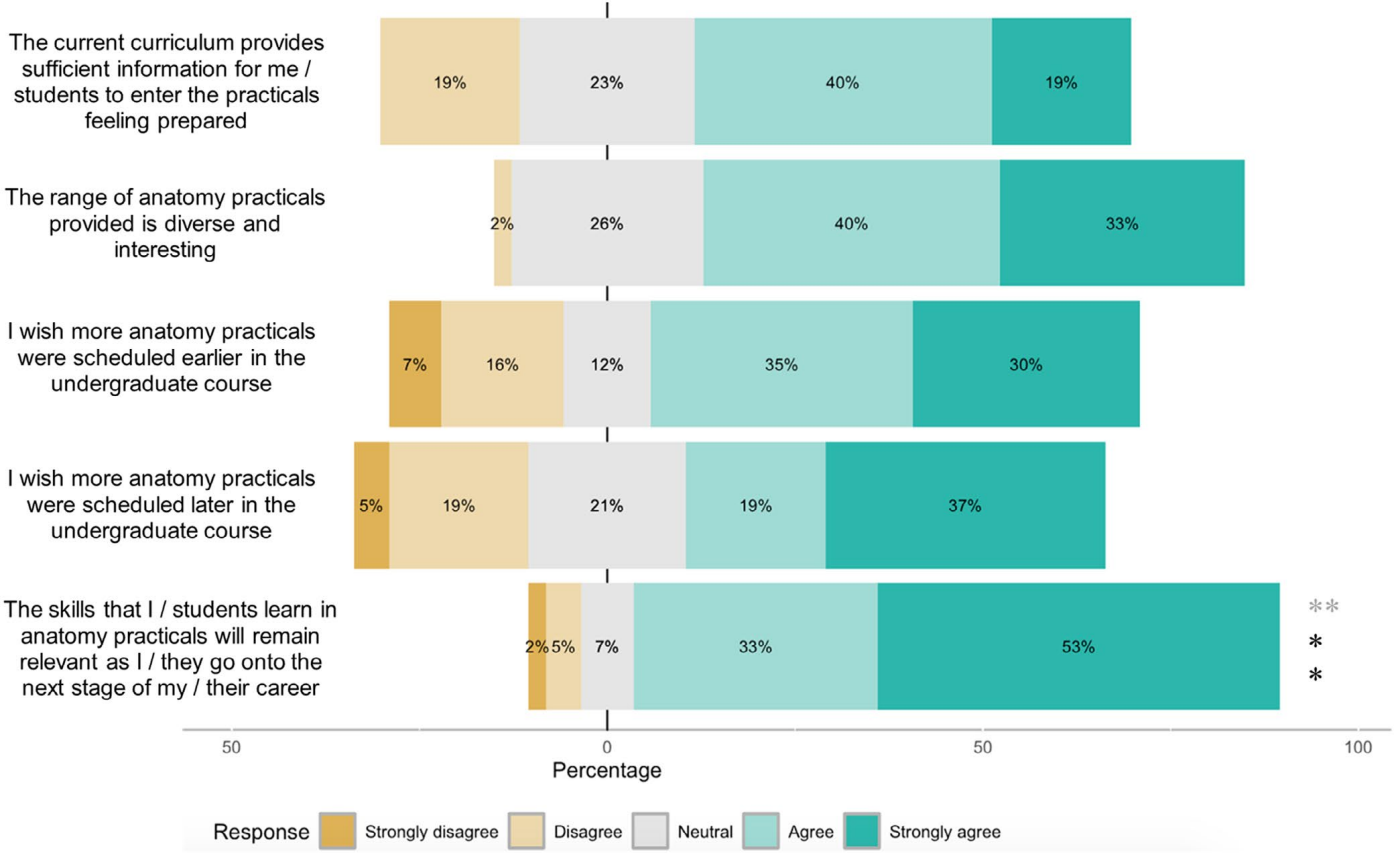
Combined survey responses to statements themed around anatomy‐based practicals, on a scale from strongly disagree to strongly agree, are reported in the order they appear in the survey. The gray asterisks indicate significant differences in how different categories of staff agreed. Those who teach anatomy practicals on veterinary medicine degrees agreed with the statement more strongly than those who teach on biology degrees (*p* < 0.01). The same is found for students when comparing responses from veterinary medicine to both biology and zoology degrees (black asterisks; *p* < 0.05; Table 3).

### The current use of anatomical models of animals in higher education

Survey participants reported that physical anatomical models (Figure 1A,B) are frequently used in practicals and teaching demonstrations, while digital anatomical models (Figure 1C) are primarily used for independent learning (Figure 8). Participants involved in biology and zoology courses reported not using both physical (chi‐squared: 22.67, *p* = 0.007) and digital models (chi‐squared: 26.47, *p* = 0.033) significantly more often than those in the veterinary courses (Table S2). When asked to rate existing digital models (i.e., institutional libraries, SketchFab, IVALA Learn, Virtual Animal Anatomy), most participants had not used them (75%), with six participants reporting in the open‐text question that they were not aware of their existence or that they did not have access to them.

I do not know where to access them, and we have not been directed to by lecturers. [Student, biology]

I find it hard to pay for something that I don't know how much I'll use. [Student, veterinary medicine]

I am neurodivergent and it's hard for me when there's so many new things and new information to catch up on. I just don't have time to go look at [digital models], especially since I feel like the digital models don't help me particularly and I would do better with going to a museum. [Student, veterinary medicine]

The participants who had previously used the DAMs suggested in the survey rated them relatively positively in the Likert scale question (Table S2). Institutional libraries were the only group that received any negative responses (11%), while the majority of participants felt neutral about how well SketchFab supported their understanding of anatomy (57%). The open‐text responses revealed both positive and negative aspects of the DAMs that explained the reasoning behind the ratings:

[DAMs from the institutional library] are useful because they are 3D so allow me to look all around the animal and identify individual structures and how they fit in as a whole. [Student, veterinary medicine]

We can print [DAMs from the institutional library] out so students can handle material that is too fragile or rare to be handed out. [Staff, biology]

**IVALA** isn't the most comprehensive and **lacks a lot of large animal resources**, it also looks fake, but being able to **toggle elements on and off is great**. **Sketchfab** is great for osteology but lots of non‐anatomical stuff so tend to **provide students with specific links** as opposed to letting them loose! [Staff, veterinary medicine]

Some are **expensive**, but these are **difficult to use in the dissection room**. [Staff, veterinary medicine]

**FIGURE 8:**
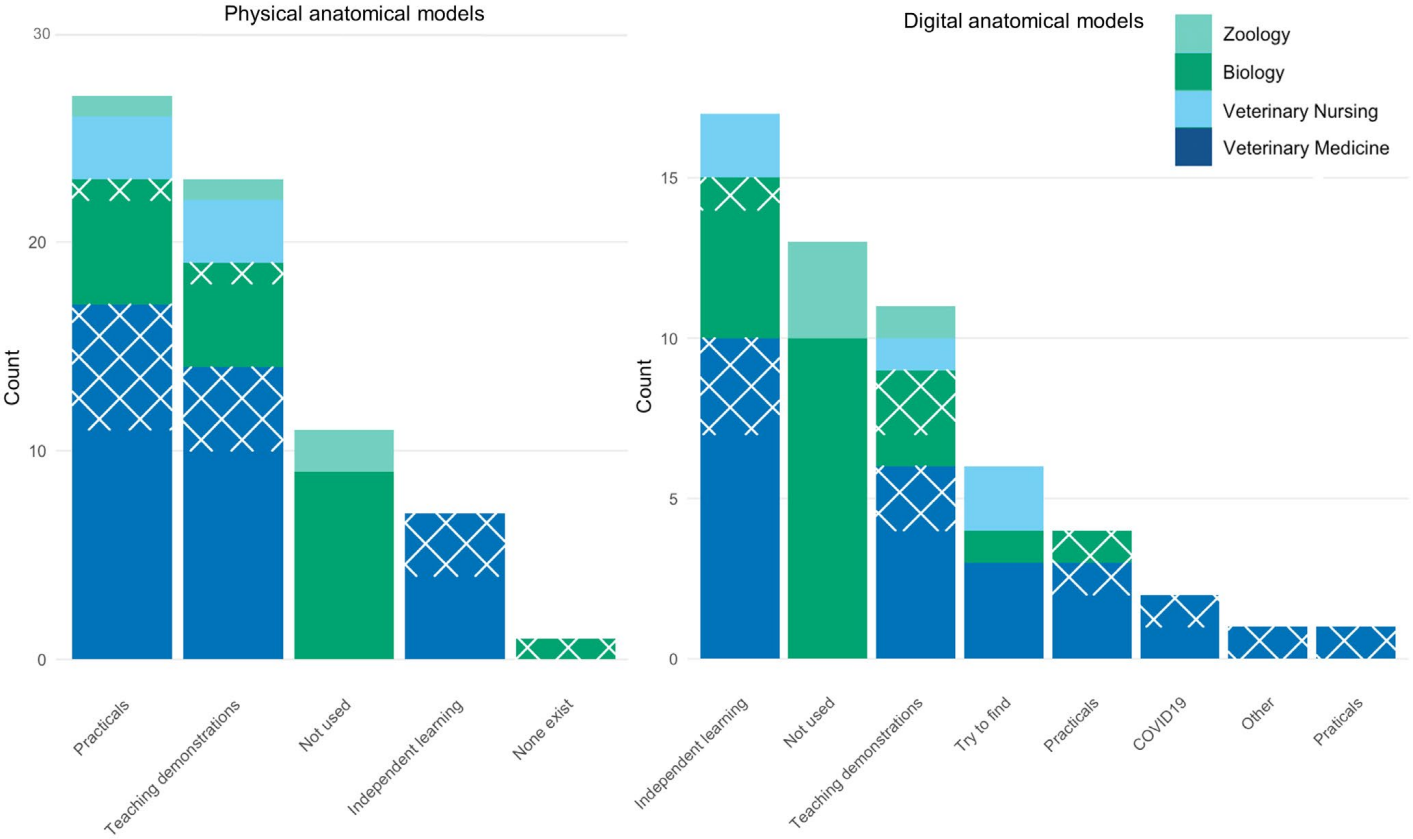
Stacked bar plots representing the ways in which physical and digital models are used to facilitate the learning of animal anatomy, color‐coded by participants' degree subject. Crosshatch patterns represent the responses from staff. “Try to find” refers to where the digital anatomical models have not been used, but now that participants have been made aware of several that exist, they will attempt to find one to facilitate future learning. “COVID‐19” refers to the response that digital models were only required during the pandemic, when face‐to‐face learning was not possible.

### The ideal digital anatomical model

Survey participants were asked a series of questions that would provide insight into which animals future researchers should prioritize making digital models for, and what the most important features are to include in a digital model. In general, responses were split fairly evenly across participants when it came to feedback on creating the ideal library of digital anatomical models (Figure 9). Access to a highly detailed part of an animal body was preferred slightly over a lower quality model of an entire body (56%). While most participants were neutral on the topic, first‐year students showed a significantly higher preference for a digital model of the entire juveniles over part of an adult specimen compared to second‐year students (Table 3). Participants who agreed that they find it easy to understand how one small anatomical component fits into the wider system were significantly more likely to show preference toward the first option for all three statements, indicating that they prefer whole‐animal models with higher levels of detail (Figure S1).

**FIGURE 9:**
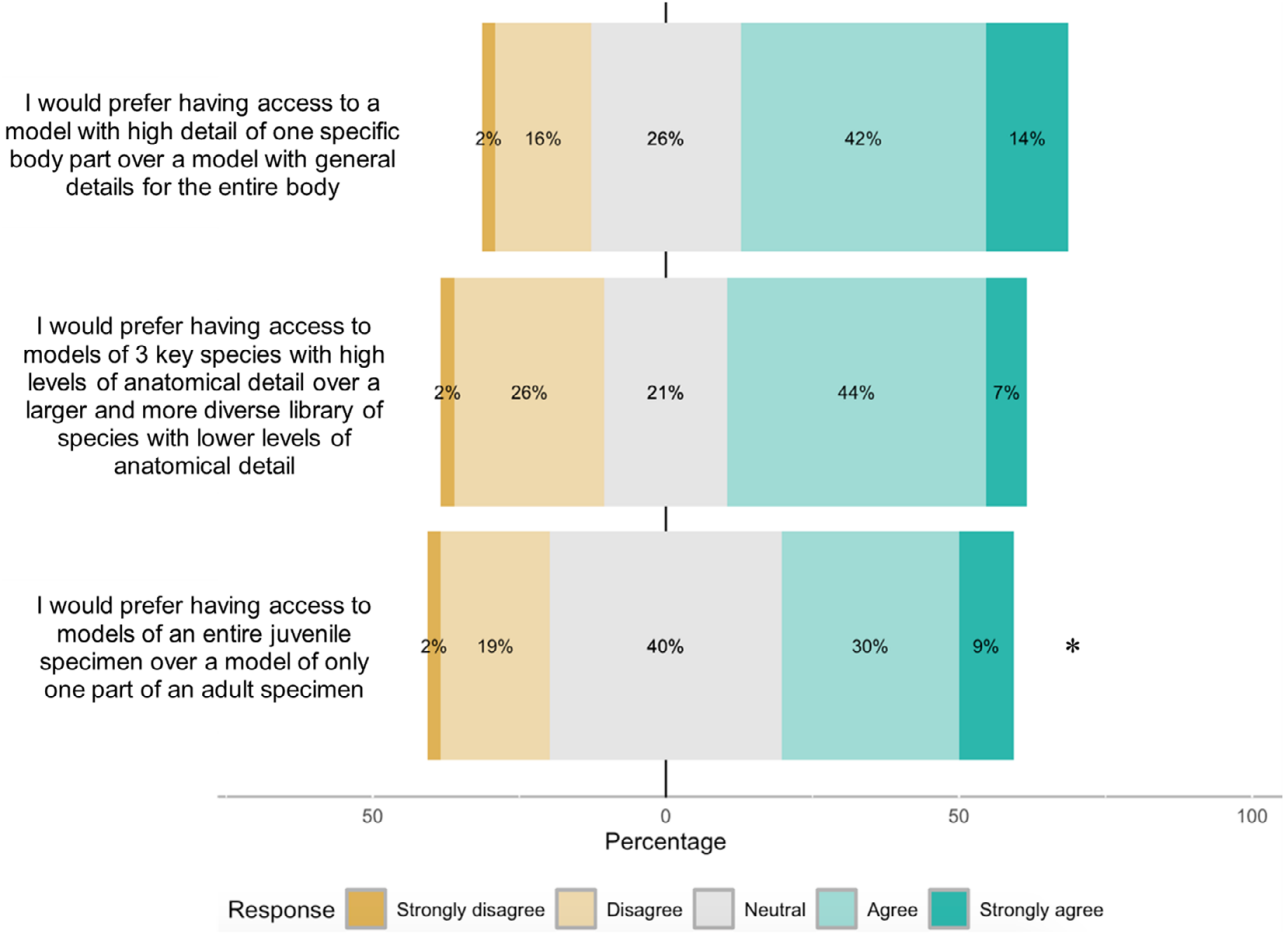
Combined survey responses to statements themed around creating the ideal library of digital anatomical models, on a scale from strongly disagree to strongly agree, are reported in the order they appear on the survey. The asterisk represents a significant difference in how first‐year students agreed more with the statement than second‐year students (*p* < 0.05; Table 3).

Participants were required to vote for the top three most important general features of the digital models—this was primarily that they had to be free to use, highly detailed, and easy to navigate (Figure 10). Having the ability to 3D‐print or access the models offline was only considered more important by three participants. There was a significant difference between degree courses for this question (chi‐squared: 26.47, *p* = 0.042), with veterinary nursing students reporting a preference for accessibility features, and veterinary medicine students voting more often for the model to be available offline (Table S2). The top three most important interactive features included the ability to turn annotations and realistic surface views on and off, animations of biomechanical function, and accompanying quizzes to test anatomical knowledge (Figure 10), while VR capabilities were the least popular feature. Respondents highlighted several other crucial features not provided in the multiple‐choice options, primarily related to smooth model rendering (*n* = 2), links to wider resources (*n* = 2), and additional interactivity features:

**Quick to load and update** ‐ it's frustrating in terms of teaching when manipulating a model is very slow. [Staff, biology]

Ability to explode the model in 3D space … to **see individual components singularly and when connected** to the other structures). [Student, zoology]

Ability to click on a specific part and **learn about not just the structure itself** but also innervation, blood supply etc and clinical relevance. [Student, veterinary medicine]

When asked which animals would be most beneficial to have as a digital model, 40 were mentioned, with dog, horse, cat, and fish as the highest scoring, non‐human animals (Figure S5).

**FIGURE 10:**
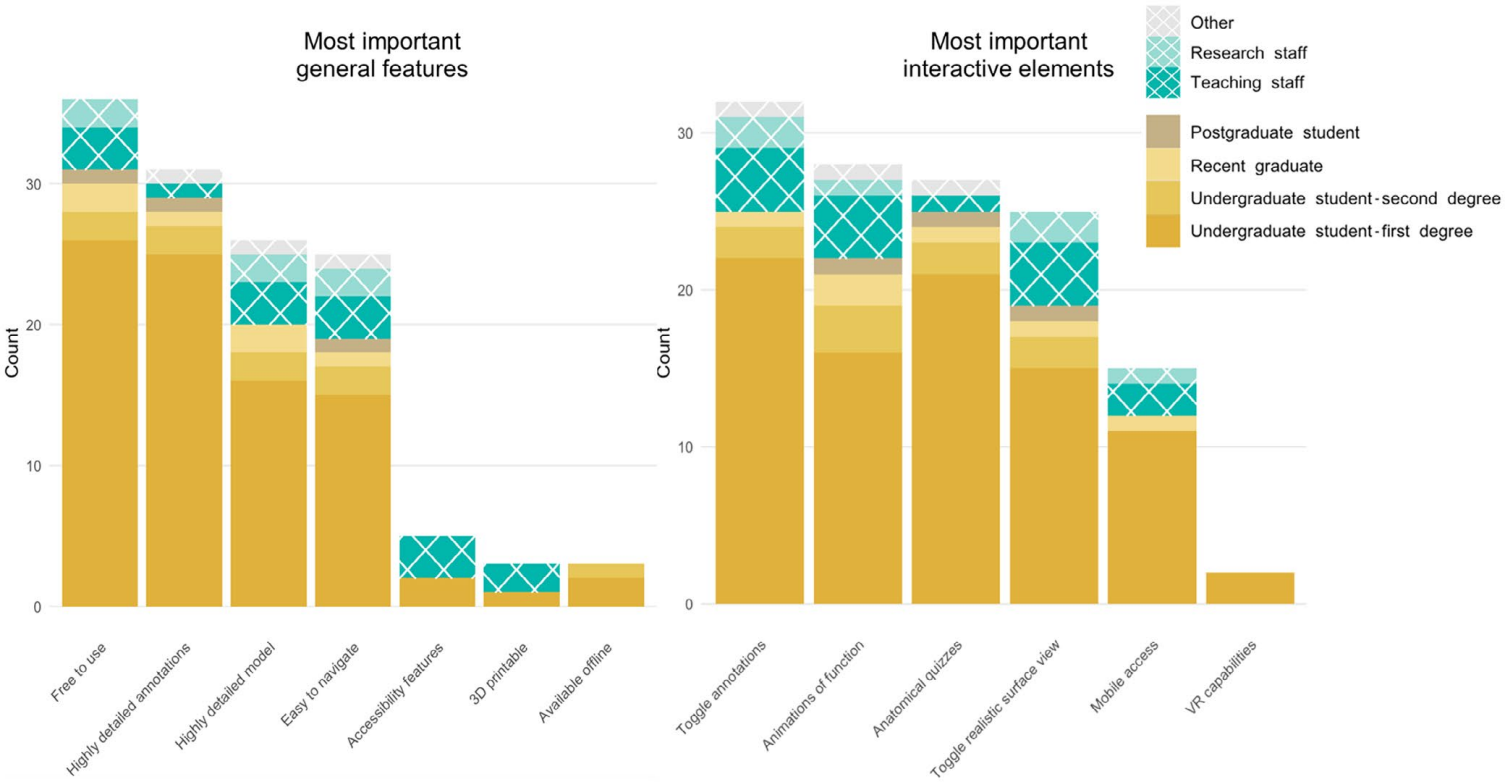
Stacked bar plots representing responses to the multiple‐choice question where survey participants had to vote for the top three most important general features and interactive elements that the ideal digital model would need to better facilitate the learning of animal anatomy, color‐coded by survey participant type. Crosshatch patterns represent the responses from staff.

### How digital anatomical models could facilitate future education and public engagement within the anatomical sciences

The general viewpoint on how DAMs would impact both students and the wider public was very positive, with no significant differences between different types of participants. The majority of survey participants agreed that they would better prepare students for their university course, their later career, and improve public understanding of science through both museums and veterinary practice (Figure 11). However, 82% agreed that better digital models are needed to facilitate anatomical education. All of these statements were positively associated with each other in the Kendall's tau‐b analysis (Figure S1).

My **career is dependent on my practical understanding** of anatomy. [Student, veterinary medicine]

Love using 3D models, digital and physical, but hard to access many really good ones on [a] **restricted HE school budget**. Also **challenging to create in‐house** without imaging facilities or the time to do it well! [Staff, veterinary medicine]

When asked to comment specifically on the implementation of more DAMs to supplement lectures (Figure 12) and traditional dissection practicals (Figure 13), most of the responses were positive. To summarize, most participants agreed that digital models would not be distracting within a teaching class setting, and that they would prove useful for exam revision, completing the tasks set in a practical, and relying less on fellow students and, to a lesser extent, teaching staff. However, it is worth noting that the majority also agreed that replacing any lectures (Figure 12) or practicals (Figure 13) with “virtual dissection labs” would be disappointing, regardless of degree subject, year group, or participant type. Seven participants made additional comments to reinforce this statement:

Digital dissection would be a great revision and/or preparation practice, but **nothing (in my opinion) would replace an experience with real specimen**. [Student, biology]

There needs to be a **hybrid approach** ‐ some digital to compliment the physical dissection. [Staff, veterinary medicine]

**FIGURE 11:**
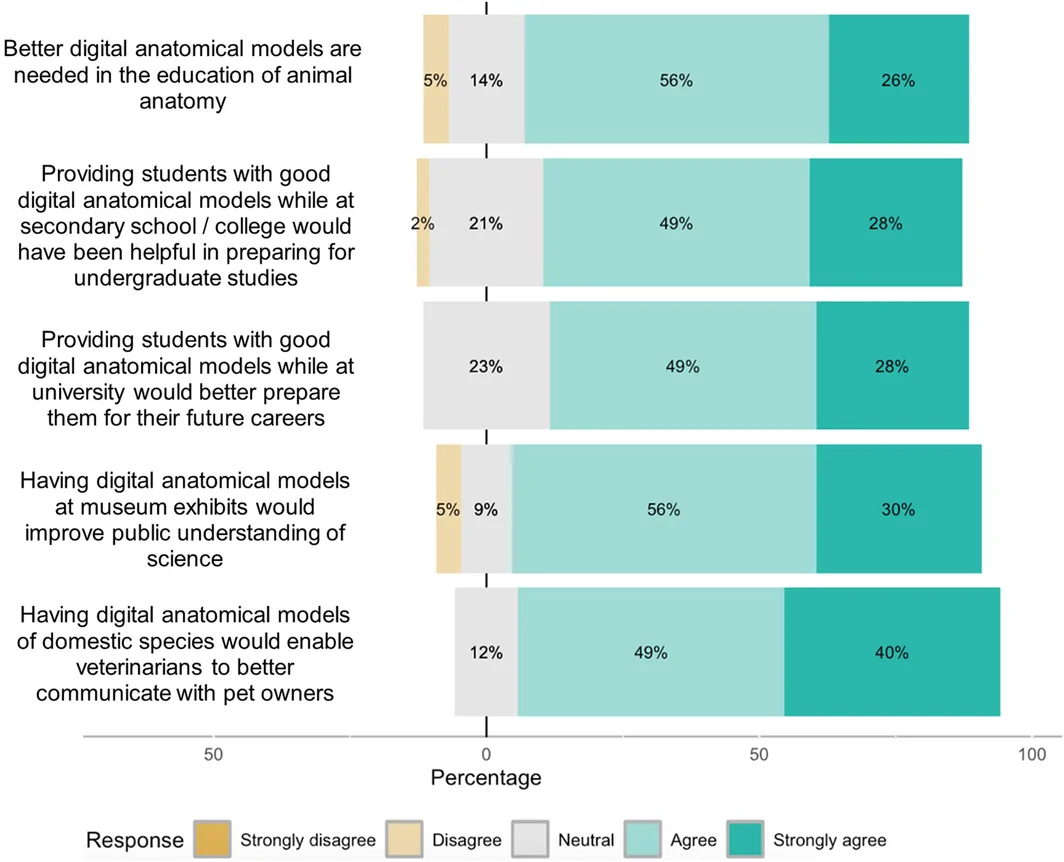
Combined survey responses to statements themed around the potential benefits of implementing better digital anatomical models, on a scale from strongly disagree to strongly agree, are reported in the order they appear on the survey. There are no significant differences in responses for any of the independent variables examined in this study for both students and staff.

**FIGURE 12:**
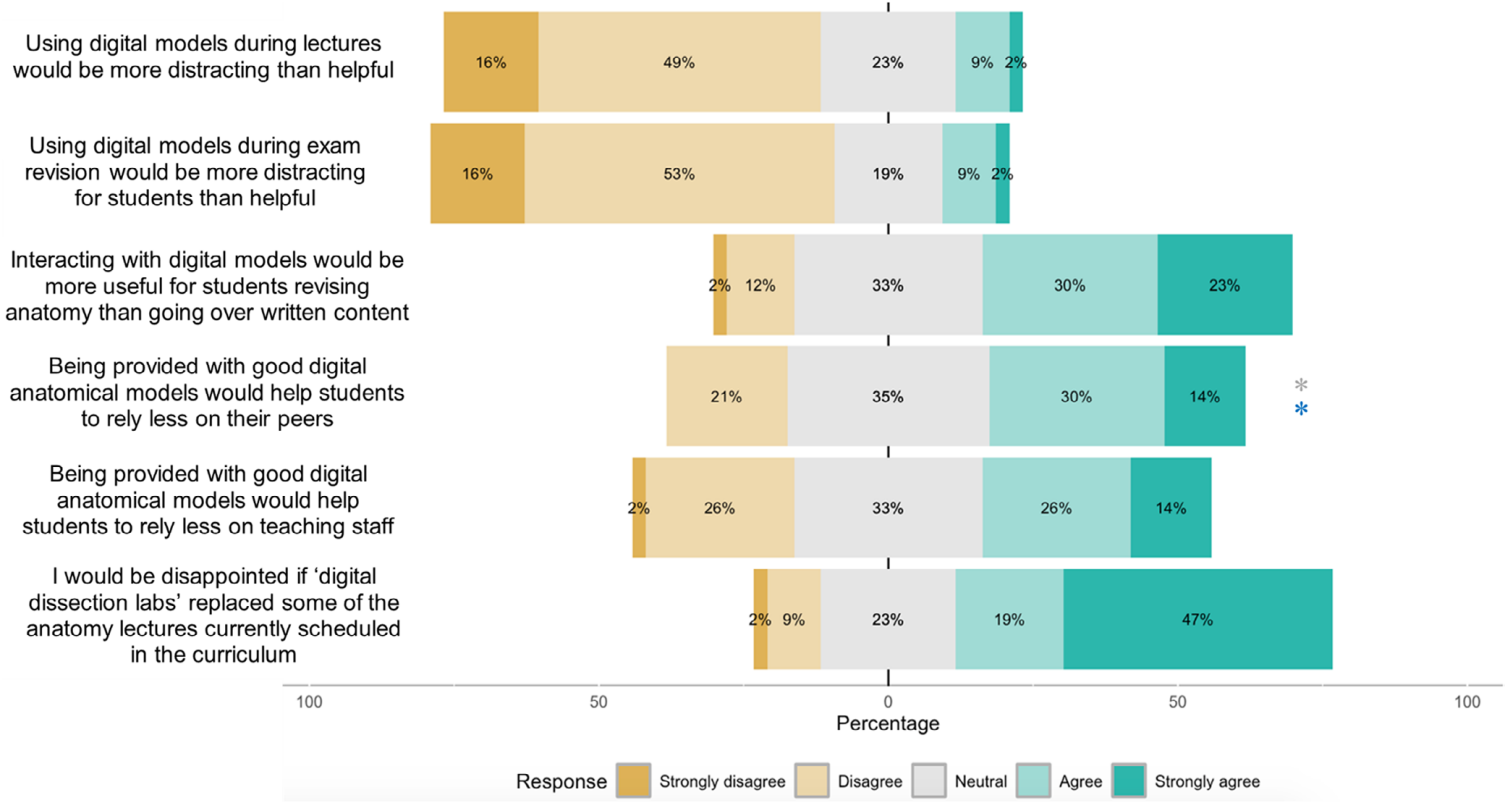
Combined survey responses to statements themed around the use of digital anatomical models in relation to lectures and exams, on a scale from strongly disagree to strongly agree, are reported in the order they appear in on the survey. The asterisks indicate significant differences in how staff who teach biology agree more with the statement that digital models would help students to rely less on their peers (gray asterisk, *p* < 0.05), but that students also agree with this statement more than staff (blue asterisk, *p* < 0.05; Table 3).

**FIGURE 13:**
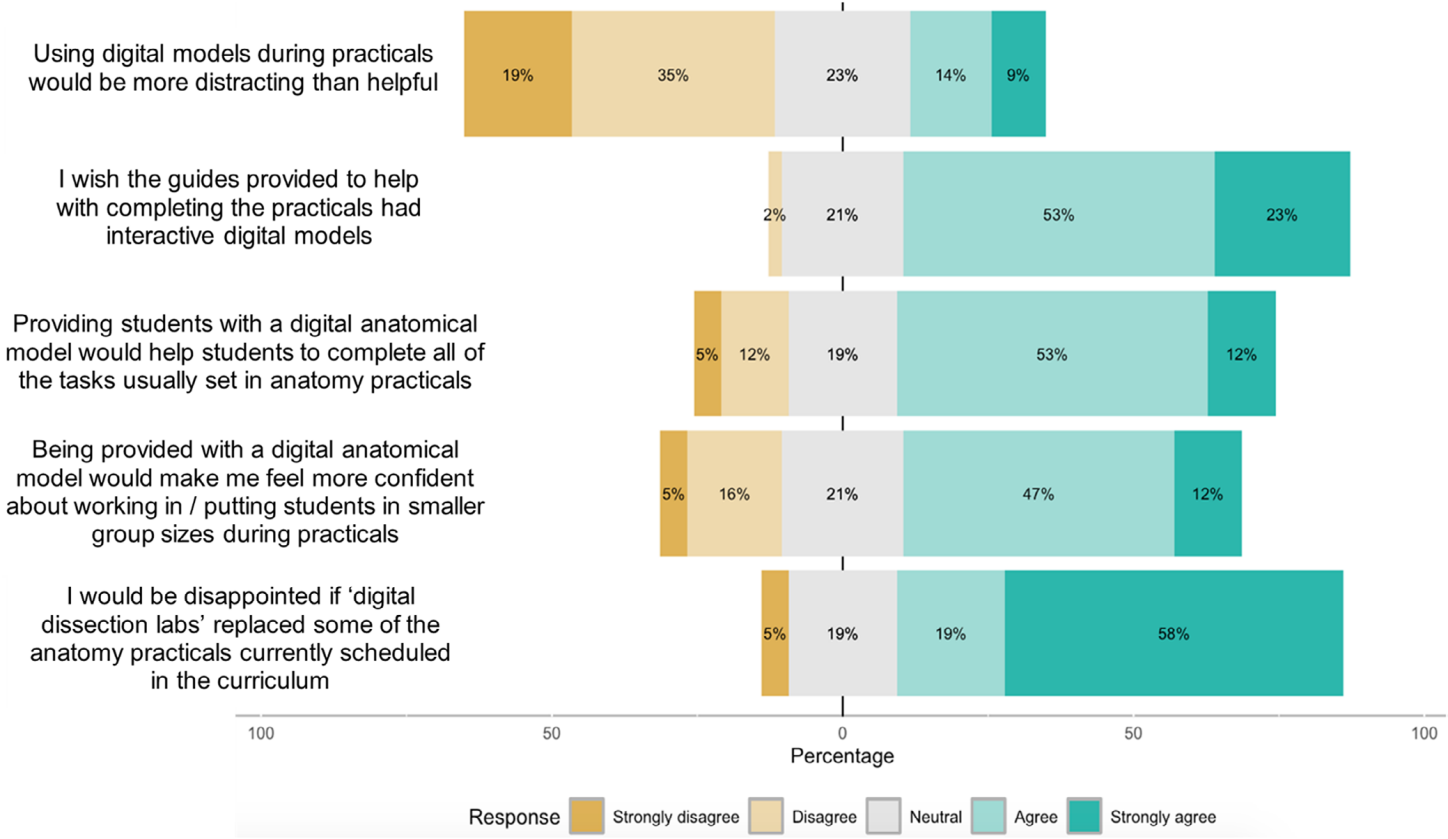
Combined survey responses to statements themed around the use of digital anatomical models in relation to anatomy practicals, on a scale from strongly disagree to strongly agree, are reported in the order they appear on the survey. There are no significant differences in responses for any of the independent variables examined in this study for both students and staff.

## DISCUSSION

The gradual integration of DAMs into higher education across the medical sciences reflects broader advancements in 3D imaging, model creation, and teaching technologies. They are frequently employed to enrich blended learning environments and provide a more multimodal curriculum, enhancing student engagement and fostering higher learning outcomes.^14^, ^21^ However, systematic reviews of the literature and the results of this survey indicate that this integration currently lags behind in the education of biological and veterinary sciences.^4^, ^12^, ^22^, ^23^ Here, we summarize what the implementation of these value resources could mean for the future of animal anatomy education and discuss the challenges that need to be overcome to facilitate this.

### University students would likely benefit in several ways from increased integration of DAMs into teaching curricula

Previous studies have shown that DAMs help students to identify anatomical structures and apply anatomical knowledge, solidifying understanding of spatial relationships and promoting the development of clinical reasoning more effectively than relying solely on written content.^6^, ^12^, ^22^, ^24^, ^25^ Therefore, it is likely that the creation and implementation of animal DAMs would help our survey participants who find it difficult to understand how structures move in 3D space (54%) and fit into the larger anatomical system (61%; Figure 2). The creation of DAMs covering a wider range of species and anatomical systems will be needed to address the difficulty associated with variation in anatomy between animals and the challenges associated with learning about structures that are widely distributed throughout the body (Figures 2 and 3) or encased in hard structures that are difficult to access with traditional dissection. As digital dissection is non‐destructive, creating digital models of rare or extinct species that could never be physically dissected would also broaden the student experience of biological diversity.

Adding virtual dissection labs to the curriculum could provide a solution to those who wish to have more anatomy practicals and those who are averse to physical dissections without significantly increasing animal use and use of lab space. The integration of DAMs in the curriculum could also help students to rely less on their peers (Table 3; Figure 12) and complete more of the practical tasks with the confidence to work in smaller groups (Figure 13). Furthermore, most traditional dissection practicals require students to share whole specimens among large groups (4–8 students) or for specimens to be split into parts (e.g., forelimbs and hindlimbs) for smaller groups (e.g., <4 students) to have a chance to perform physical dissections (AL, personal observation). Virtual dissection classes are only limited by computer availability, and may therefore result in a more individual experience for each student. Many previous studies which have quantified the impact of a DAM on the student experience using educational intervention support these responses—participants reported increased engagement, spatial understanding, confidence, and, in many cases, performance in tests of anatomical knowledge (animal DAMs^18^; human DAMs^8^, ^9^, ^13^, ^15^, ^21^, ^25^; but see O'Reilly et al.,^10^ Saltarelli et al.,^26^ Vaduva et al.^6^).

DAMs can also be used as Supporting Information to support current anatomy practicals. Embedding them within the guide provided alongside a traditional dissection has been identified as a useful place for technological integration (Figure 13). Additionally, DAMs are currently used more often prior to practicals as a form of preparation and could be better integrated into the consolidation of anatomical knowledge after the practicals take place (Figure 6). Quizzes could involve applying what was learned in the traditional dissection to the virtual body. If used as revision materials, DAMs would provide the flexibility of being able to effectively repeat the practical at the students' own pace, undo errors, and repeatedly revisit complex structures, which is a considerable advantage over organizing more frequent physical dissections. This would help to reinforce the many skills learned in anatomy practicals that better prepare veterinary students for their future careers.

### What is the ideal DAM?

The results of this survey present a list of guidelines for the creation of future high‐quality DAMs that will best facilitate future research and education. While the ideal DAM would include all the interactive elements identified in the survey, participants viewed the ability to toggle on and off annotations and different surface views, observe animations of function, and complete accompanying quizzes to test knowledge as the most important (Figure 10). Participants also emphasized that they must be free to access for all, highly detailed, and easy to navigate (Figure 10). Previous studies have also highlighted that spatial realism, resolution, and interactivity—the ability to manipulate structures in isolation, zoom, rotate, and see detailed anatomical labels—are crucial to achieve learning objectives and support active exploration.^11^ Incorporating these in all future models will improve the current lack of consistency in the formats and methods used to create and distribute DAMs, which can lead to difficulties in integrating these tools across different educational platforms.^23^

It is also crucial to consider accessibility. Not only should DAMs be accessible across a variety of devices and languages,^15^ but also reflect the requirements of diverse communities—a third of survey participants reported a form of disability or neurodivergence. The ideal DAM would have color‐blind friendly palettes and audio description of annotations, for example. Additionally, DAM creators should aim to provide lower‐resolution versions of the models to account for differences in computing power between institutions to ensure equity in anatomical education, especially in developing regions.^8^ Furthermore, DAMs should integrate anatomical, clinical, and educational elements to support a range of pedagogical goals, from knowledge acquisition to skills development.^7^ Previous literature has emphasized that the models should be created with relevant curricula and assessment strategies in mind, with measurable learning outcomes.^26^ Combining all these features into a comprehensive, open‐access DAM with the broader educational framework in consideration could transform how animal anatomy is taught, from secondary schools to universities and public museums.

### What might be preventing the use of existing DAMs?

Most survey participants responded “NA” to opinions on how well the existing DAMs we presented in the survey supported their learning/teaching (Figure S4), indicating that they were not aware of them, or had not used them. This was most common outside of DAMs present in institutional libraries. Several participants mentioned aversion due to cost and lack of relevant taxa. However, there are many high‐quality DAMs which are generated through the investigation of biological research questions and rising efforts to digitize museum specimens^27^, ^28^, ^29^, some of which even come with lesson plans^30^. Unfortunately, the leading platform that comparative anatomists use to share DAMs—Sketchfab—is converting to a marketplace, making the future of accessing free high‐quality resources uncertain. Having open‐access resources compiled in one place as a digital anatomical library is crucial for effective dissemination. It is crucial that persistent URLs are used to ensure the long‐term accessibility of these models—some research‐based atlases we found during our review of the literature were not readily available.^31^, ^32^, ^33^

There are also technological challenges to be considered. High rendering demands and platform incompatibility can hinder the user experience,^24^ and there needs to be enough devices for small groups of students to be able to examine existing models as a class. It is also important to provide students and educators with sufficient training to tackle the learning curve associated with complex 3D models, especially if they lack prior exposure to digital tools.^6^ Orientation sessions, digital skills training, and ongoing support are required for students to achieve their learning objectives.^12^ The Non‐Clinical Tomography Users Research Network (NoCTURN) Education & Outreach community has recently begun producing education kits, which will help integrate biomedical imaging and the DAMs they generate into STEM education, such as their Introduction to MorphoSource.^34^

While we have discussed the many benefits of DAMs and encouraged a raised awareness of resources available, our survey raises an important point to consider before implementing them into teaching. Most participants reported that they would be disappointed if their current anatomy lectures and practicals were replaced (Figures 12 and 13). In the open‐text responses, many emphasized that virtual dissection labs should by no means become a total substitution for traditional teaching approaches. Previous studies have highlighted concerns that heavy reliance on digital technology without complementary hands‐on experience could reduce the motor skills and tactile experience required for traditional dissections.^10^, ^12^, ^35^ It may be that striking the balance between virtual and physical teaching is difficult and could be limiting the use of existing DAMs. There are, however, several UK universities that teach human medicine, which do not use body donors.^16^ Further research into how these institutions approach this challenge would be beneficial for exploring the integration of DAMs into the teaching of animal anatomy on biological and veterinary science degrees.

### Challenges facing the creation of more high‐quality DAMs

Despite the considerable number of participants who were not aware of existing DAMs, the vast majority still recognized the benefits they could bring to their learning and the general public (Figure 11). There are several challenges associated with the generation of new animal DAMs. First, it is crucial to consider the ethical implications surrounding the creation of resources derived from body donors, obtaining consent from pet owners and ensuring anonymity is maintained. In the same way that there should be variation in human bodies for medical teaching,^12^ animal DAMs should cover a range of breeds and species—survey participants reported that the relevant taxa are not currently available as a DAM (Section 3.2). However, as with teaching using donor materials, DAM creators are limited to what material is available to scan.

High‐quality DAMs are costly and time‐consuming to create. If possible, DAMs should be derived from CT and MRI scans, rather than surface scans. These approaches allow detailed imaging of complex objects at a high resolution without destroying the 3D structure of the specimen.^7^, ^28^, ^29^ Individual anatomical structures can be isolated, meaning models can be rigged with animations that demonstrate biomechanical function, and students would have the ability to digitally dissect specimens, which is impractical with surface scans. However, this requires specialized and expensive equipment that is not readily available at many universities, while surface scans can be performed with smartphones.^36^ Every researcher in the field of anatomical sciences who is already generating scan data for other purposes should aim to convert them into accessible educational resources. This would greatly contribute toward the need for more comprehensive, species‐specific models to enhance veterinary and biosciences education without additional cost to educators. It is increasingly important to publish the anatomical models and corresponding imaging data of study animals online in open‐access repositories (e.g., MorphoSource) to facilitate future research and education. Free software and corresponding tutorials exist to facilitate the transformation of biomedical imaging data to 3D digital models, such as Blender and 3DSlicer.^7^ Furthermore, we recommend that the time and resources needed for interactive digital model creation and publication should be included in future grant proposals where possible.

### Study limitations

The latest estimate in population size of veterinary students across all 10 UK universities was from 2021–2022 (8669), attained from the latest publication of RCVS Facts (2022). According to this value and a Qualtrics calculator using a confidence level of 95%, the ideal sample size for this survey was 368 participants. This does not include teaching staff nor biosciences students—the Higher Education Statistics Agency provides the total number of university students studying “biological and sports sciences,” but it is not possible to discern the number of these students who are provided animal anatomy practicals as part of their curricula. As such, the sample size achieved by this survey is small, despite our best efforts to provide incentives and distribute the survey as widely as possible. Incentivization risks participant anonymity, so it can be difficult to achieve both high response rates as well as anonymous participation, without physical incentives that can be distributed in real time (impossible for a geographically dispersed survey population). We were keen to capture the views of as many participants as possible from a diverse community across several universities. As a result, we could not accurately report the absolute response rate. Ultimately, the statistical power, while sufficient for the study, is problematic for the population sample, thus limiting the generalizability of the results of this study to the broader population. As such, any attempt to generalize from these results to a wider population context should be done with caution. This research, however, still provides novel insights into the importance of DAMs in facilitating learning in animal anatomy, and how greater efforts are required to share and create them across a broader range of taxa spanning veterinary and biological science curricula.

## CONCLUSION

This study has provided a comprehensive overview of how digital models are currently used to teach and learn animal anatomy in veterinary and biosciences degrees across the UK. We found a notable lack of awareness of many existing DAMs—research into standardization across these resources could help facilitate their wider adoption and interoperability across degree courses. Additionally, while beyond the scope of this current study, testing multiple existing DAMs via educational interventions (e.g., computer‐based anatomy practicals) could directly quantify how they impact learning, where implementation into the curriculum is most suitable. Running this kind of study over several cohorts will provide much‐needed evidence regarding the long‐term impact of these tools,^6^, ^15^ especially whether they help in acquiring deep anatomical knowledge.^25^ Researchers and educators can use the results of this survey as a checklist of features to include in DAM creation to maximize the impact and accessibility of their models. Our analysis also indicated that while high‐quality DAMs hold the potential to transform anatomy education, this would work most effectively via a hybrid approach with traditional anatomy practicals. This would enable more equitable access to high‐quality resources to support consolidation of anatomical knowledge. Addressing the challenges facing the development of high‐quality digital libraries, such as technological limitations and funding constraints, will require coordinated efforts between anatomical researchers, educators, and public‐facing institutions.

## AUTHOR CONTRIBUTIONS

**Sarah Channon:** Methodology; validation; investigation; writing – review and editing. **Alice Leavey:** Conceptualization; methodology; data curation; investigation; formal analysis; funding acquisition; visualization; project administration; resources; writing – original draft; writing – review and editing.

## FUNDING INFORMATION

This study did not receive any external support—survey incentives were self‐funded.

## CONFLICT OF INTEREST STATEMENT

The authors have no conflicts of interest to declare.

## ETHICS STATEMENT

Ethical approval for the survey was obtained through UCL's central Research Ethics Service (Ethics ID 28615/001).

## ARTIFICIAL INTELLIGENCE DISCLOSURE (AID) STATEMENT

AI tools were not used in the research or in the preparation of this manuscript.

## PERMISSION TO REPRODUCE MATERIAL FROM OTHER SOURCES

No permissions were required.

## Supporting information


**Data S1.** Supporting Information.

**Appendix A.** Student survey
**Appendix B**. Staff survey.
**Table S1**. Results of the Cronbach's alpha test of reliability for each theme in the survey, where negatively worded statements have been reverse‐coded to ensure the same directionality between statements. “Challenge data” refers to where participants were asked to rate how challenging each area of the body is to teach/learn. Several areas were non‐applicable to many participants, so we also analyzed Cronbach's alpha for the theme of general experiences with anatomy, with and without these data.
**Table S2**. The result of the chi‐squared tests that showed significant differences in the answers to multiple‐choice questions between predictor variables. The staff survey responses showed no significant differences.
**Figure S1**. The results of Kendall's Tau‐b analysis, which compares the strength of association between each pair of Likert scale questions. Blue tones represent a significant positive relationship, red tones indicate a significant negative relationship, and blank boxes indicate no significant relationship. The question asking participants to rate how useful the digital anatomical models are on SketchFab had to be removed due to the presence of too many NAs. Note that the questions have been shortened/amended to fit within the plot space—full versions of each question can be found in Appendices A and B.
**Figure S2**. Participant views about how they find specific aspects of their current anatomy practicals. The black asterisk represents a significant difference in how students interpreted the safety of their anatomy practicals. First‐year students agreed more strongly that their anatomy practicals are safe than second‐year students (*p* < 0.05; Table S2).
**Figure S3**. A summary of the number of times each response was mentioned for the open‐text question: *For which animals do you find the anatomy most difficult to learn/teach?* The responses are color‐coded by the categories “general” (yellow), “large animal group” (green), “specific animal” (orange), “anatomical system” (red), and “biomechanical” (gray).
**Figure S4**. Likert scale ratings of existing digital anatomical models. These data could not be statistically analyzed due to low sample size (total number of participants = 43), as many staff and students had not previously used or were not aware of these resources.
**Figure S5**. A summary of the number of times each response was mentioned for the open‐text question: *Which animal(s) would students benefit from most by having a digital anatomical model?* The responses are color‐coded by the categories “general” (yellow), “large animal group” (green), and “specific animal” (orange).

## Data Availability

Collective, anonymized data and results of statistical analyses are available in the Supporting Information.
