# Supplementary material for: Student and staff experiences of interactive digital models when learning animal anatomy
Source: Anat Sci Educ. 2026 Jun 11;19(10):1577–96. doi: 10.1002/ase.70286 (PMC13654050; doi:10.1002/ase.70286)
Supplement: Supplementary file 1 [file ASE-19-1577-s001.docx]

Supplementary Information for Leavey, A. & Channon, S. Student and staff experiences of interactive digital models when learning animal anatomy.

**Appendix A - Student survey**

The purpose of this study is to investigate how interactive digital models have been used to facilitate the learning of animal anatomy at university level. This online survey is primarily designed for veterinary and biosciences students who have participated in at least one in-person anatomy practical as a part of their course. This can include postgraduate students and recent university alumni (graduating in 2021 and onward) who would like to share their experience. We are keen to capture the views of students from across a diverse community across several universities.

The areas that we are inviting you to respond to are:

- Section 1: About you.
- Section 2: Experience learning anatomy so far.
- Section 3: Your anatomy practicals.
- Section 4: Your use of digital anatomical models so far.
- Section 5: What makes the ideal digital anatomical model?
- Section 6: How digital anatomical models impact your learning.

Please read all of the questions and statements carefully.
 **What do you mean by…?** 
When referring to ‘anatomy practicals’, we are primarily discussing the physical dissection, manipulation and/or handling of specimens, whether fresh, fixed (i.e., preserved in formalin), plastinated, skeletonized, fossilized, or as 3D printed replicas. This can include practicals where specimens are handled by only staff, including postgraduate teaching assistants, where students simply observe and take notes. Anatomy practicals can take place in multiple settings (e.g., lab, museum, zoo). 
‘Physical anatomical models’ refer to fixed specimens (Figure 1A) and 3D prints (Figure 1B). In veterinary schools, these are usually found around your building. ‘Digital anatomical models’ refer to interactive 3D structures that can be viewed and manipulated on websites or apps (e.g., SketchFab.com; Figure 1C). Digital models do not include static 2D images on a PowerPoint, for instance. They usually have accompanying annotations and sometimes animations. 

**What's in it for me?** 
It is anticipated that the survey will provide valuable information that will help us to enhance students’ experiences on veterinary and biosciences programmes. There’s also a prize draw to win one of ten £5 vouchers at the end of this survey as a thank you for participating.

**The survey has been kept relatively short (estimated completion time is approximately 15 minutes) so we hope you can find the time to complete it.**

B)

**Figure 1** - Example of the different types of anatomical models commonly used in practicals, demonstrated by the distal limb of a horse. A) a physical anatomical model in the form of a real, skeletonised specimen (image credit: Walter Varcoe); B) a physical anatomical model in the form of a 3D-printed model (image credit: 3D VetAnatomize); C) an interactive digital anatomical model created from a surface scan of a real skeleton (credit: University of Dundee Museum Collections; sketchfab.com).

C)

A)

**Data protection privacy notice** 
The controller for this project is University College London (UCL). The UCL Data Protection Officer provides oversight of UCL activities involving the processing of personal data. If you are concerned about how your personal data is being processed, or if you would like to contact us about your rights, please contact UCL in the first instance at data-protection@ucl.ac.uk 

The information that is required to be provided to participants under data protection legislation (GDPR and DPA 2018) is provided across both the ‘local’ and ‘general’ privacy notices. This ‘local’ privacy notice sets out the information that applies to this particular study. Further information on how UCL uses participant information can be found in our ‘general’ privacy notice https://www.ucl.ac.uk/legal-services/privacy/ucl-general-research-participant-privacy-notice. 

All data will be handled and stored safely according to the General Data Protection Regulations. The lawful basis that will be used to process your personal data, including any special category data is: ‘public task’ and ‘research purposes’. All personal data you provide will be anonymised and we will endeavour to minimise the processing of personal data wherever possible. We will only share findings from this survey in a way that ensures no data will be attributable to an individual, presenting statistical data in the aggregate (collectively). All quotations will be anonymised in any write up. No raw data will be shared with any third parties - only the researcher of this project will have access to datafiles containing potentially identifying information. 

Dr Alice Leavey (Centre for Integrative Anatomy, UCL) is the researcher conducting this survey and performing all data analyses. You can contact her at ucbtal9@ucl.ac.uk if you have any questions about the project. This research has been approved through the central Research Ethics Service at UCL (Ethics ID number 28615/001).

**Q1. I have read and understood the privacy notice.**

Please note: answering 'No' means you will not be able to proceed with the survey.

- Yes / No

**Do I have to provide any personal data?** 
The final section of this survey invites you to opt in to future focus groups which intend to get your feedback on the digital models once they have been made, as well as the opportunity to win a prize for completing the survey. To enter you would need to submit your email address - this is the only identifiable information being collected in this survey and this step is entirely optional. You do not have to provide your email address. Email addresses submitted only for the prize draw will be deleted as soon as the winners are rewarded (approximately one month after the survey deadline). Email addresses submitted for the focus groups will be deleted as soon as these have taken place (approximately three years following this survey depending on the timing of further funding), or as soon as it is decided that the focus groups will no longer go ahead. 

The only special category data you will be asked to provide as part of this survey will be indicating whether you have any additional needs that would need to be considered when creating the digital models (e.g., colour blindness, physical disabilities, neurodivergence). Participants are provided with the option ‘Prefer not to say’, so you do not have to provide this data if you do not wish to.

Consent to participate in this survey will be assumed throughout the completion of the survey. Please note that your participation is completely voluntary, and you can withdraw at any time before submitting the survey. Any incomplete survey responses will be deleted. Once the survey deadline has passed, the anonymous data you provide will be processed only for as long as it is required to complete the wider research project, after which it will then be deleted.

**Q2. I consent to taking part in this survey.**
Please note: answering 'No' means you will not be able to proceed with the survey. By answering ‘yes’, your consent will be assumed throughout the completion of this survey.

- Yes/no

## **Section 1: About you**

This section asks some basic questions about you and your current degree programme. In responding, please remember that – as with the entire survey – your individual responses will be anonymized and we will report on findings in a way that ensures no information will be attributable to any individual.

**Q3. Which best describes you? *** [select one]

If you are a member of staff, do not submit answers to this survey – instead please follow this link to the survey that is designed specifically for you: <https://forms.office.com/e/aWxhAdeHPG>

- Undergraduate student completing their first degree
- Undergraduate student completing their second degree
- Recent graduate currently not in education
- Postgraduate student
- Other: __________

**Q4. What year of your current course are you in? *** [select one]

Note: Please do not participate in this survey unless you have already participated in at least one practical on animal anatomy.

- First
- Second
- Third
- Fourth
- Fifth
- Sixth
- Other: __________

**Q5. Which of the subjects below best represents your current / most recent degree course?*** [select one]

- Biology
- Zoology
- Veterinary Medicine
- Veterinary Nursing
- Bioveterinary
- Other: ___________

**Q6. Do you have experience with anatomy practicals prior to your current/most recent degree programme?** [option to select multiple]

Going forwards, please complete this survey with only your current/most recent degree course involving anatomy practicals in mind.

- No
- Yes – during school
- Yes – during my first undergraduate degree

**Q7. Please tick all of the following categories that apply to you:*** [option to select multiple]

Please remember that this special category data - as with all the data collected in this survey - will only be reported anonymously and will not be shared with any third parties. Answering this question will be important to enable us to create the most accessible digital models possible.

- I am Neurodivergent (e.g., I have dyslexia, dyspraxia, dyscalculia or ADHD, or am on the autistic spectrum)
- I have a mental health condition (e.g., depression, schizophrenia or anxiety disorder)
- I have a neurological condition (e.g., epilepsy)
- I have a physical impairment or mobility issues, such as difficulty using arms
- I am d/Deaf or have a serious hearing impairment
- I am blind or have a serious visual impairment uncorrected by glasses
- I am colour blind (monochromatism, red-green colour, blue-yellow colour)
- I would prefer not to say
- None of the above
- Other: ____________

**Section 2: Your experiences learning animal anatomy**

This section is about how you have found learning about the anatomy of animals so far in general. Please remember to read all of the questions and statements carefully.

**Q8. Reflecting on all of your experiences learning anatomy so far, to what extent do you agree with the following statements?*** [Likert scale]

Select 'N/A' for the final statement if you have not yet taken an assessment on anatomy.

[A] Overall, I find anatomy an enjoyable topic to learn

[B] All body regions are equally difficult to learn in great depth

[C] The anatomy of all animals is equally difficult to learn in great depth

[D] I find it easy to understand how anatomical structures move in 3D space

[E] I find it easy to understand how one small anatomical part fits in with the rest of the larger system

[F] I often score poorly in assessments on topics related to anatomy

All: Strongly disagree / Disagree / Neutral / Agree / Strongly agree.

**Q9. How difficult are each of these body regions to fully understand?*** [Likert scale]

If you have not been taught the body region, select 'N/A'.

[A] Skeletal

[B] Muscular

[C] Digestive

[D] Renal

[E] Nervous

[F] Cardiovascular

[G] Lymphatic

[H] Respiratory

[I] Endocrine

[J] Reproductive

All: Very challenging / Challenging / Neutral / Easy / Very easy / Not applicable.

**Q10. For which animals do you find the anatomy most difficult to learn, and why?*** [free text]

_____________________________

**Q11. Please provide any information that you think would help us to understand your responses to the questions above. In particular, we would love to hear about specific processes, 3D structures, or biomechanical motions that are the most difficult to understand.** [optional; free text]

_____________________________

**Section 3: Your anatomy practicals**

This section includes some basic questions about the structure of your anatomy practicals.

**Q12. On average, how often are you scheduled to have anatomy practicals in an academic year?*** [select one]

- Twice a week
- Weekly
- Monthly
- Once every few months
- Twice a year
- Once a year

**Q13. What are you required to do in advance to prepare for anatomy practicals? Select all that apply.*** [option to select multiple]

- Watch a video demonstration
- Examine a physical anatomical model
- Examine a digital anatomical model
- Review lecture content
- Complete a written assignment
- Deliver a presentation
- Directed reading
- Compare notes with peers
- Complete a quiz
- Nothing
- Other: __________

**Q14. What are you required to do after anatomy practicals to consolidate learning? Select all that apply.*** [option to select multiple]

- Watch a video demonstration
- Examine a physical anatomical model
- Examine a digital anatomical model
- Review lecture content
- Complete a written assignment
- Deliver a presentation
- Directed reading
- Compare notes with peers
- Complete a quiz
- Nothing
- Other: __________

**Q15. On average, how often do you get to do the following activities during practicals?*** [Likert scale]

[A] Physically dissect a fresh/fixed specimen

[B] Physically handle a fresh/fixed specimen

[C] Physically handle a skeleton/fossil

[D] Watch a demonstrator dissect a fresh/fixed specimen

[E] Watch a demonstrator handle a fresh/fixed specimen

[F] Watch a demonstrator handle a skeleton/fossil

[G] Make notes from observing specimens that cannot be handled (e.g., behind a barrier at a museum)

All: Never / Rarely / Sometimes / Often / Always.

**Q16. So far, my anatomy practicals have been…*** [Likert scale]

[A] Engaging

[B] Interactive

[C] Valuable

[D] Safe

[E] Appropriately challenging

[F] Ethical

[G] An important part of my degree

All: Strongly disagree / Disagree / Neutral / Agree / Strongly agree.

**Q17. Are there any other skills, capabilities or awarenesses that you feel the anatomy practicals helped you to develop?** [optional; free text]

__________________________________

**Q18. Reflecting on all of your anatomy practicals so far, to what extent do you agree with the following statements?*** [Likert scale]

[A] The current curriculum (e.g., lectures, online learning material) provides sufficient information to enter the anatomy practicals feeling prepared

[B] The range of anatomy practicals provided is diverse and interesting

[C] I wish more anatomy practicals were scheduled earlier in the undergraduate course

[D] I wish more anatomy practicals were scheduled later in the undergraduate course

[E] The skills learned in anatomy practicals will remain relevant as I go onto the next stage of my career

All: Strongly disagree / Disagree / Neither agree or disagree / Agree / Strongly agree.

**Q19. Reflecting on all your anatomy practicals so far, what aspects do you feel were most beneficial for preparing you for your current / most recent degree course?** [optional; open field]

_____________________________

**Q20. Were there any aspects of your anatomy practicals which you feel could have been improved to better prepare you for your current / most recent degree course?** [optional; open field]

_____________________________

## **Section 4: Your use of digital anatomical models so far**

This section is about the digital models that you have used throughout your degree to develop your understanding of anatomy.

**Q21. How often do you use physical models, such as fixed specimens or cadavers, to learn anatomy?*** [option to select multiple]

Select all that apply. As a reminder of what a physical anatomical model is, please see Figure 1.

- I have never used a physical anatomical model before
- I have never used a physical anatomical model before, but will now try to find one
- I am required to use physical anatomical models as a part of independent learning (e.g., preparing for lectures or exams)
- I am required to use physical anatomical models as a part of my practicals
- Teaching staff use physical anatomical models for demonstrations
- Other: __________

**Q22. How often do you use digital models, such as annotated and interactive 3D models available online, to learn anatomy?*** [option to select multiple]

Select all that apply. As a reminder of what a digital anatomical model is, please see Figure 1C.

- I have never used a digital anatomical model before
- I have never used a digital anatomical model before, but will now try to find one
- I am required to use digital anatomical models as a part of independent learning (e.g., preparing for lectures or exams)
- I am required to use digital anatomical models as a part of my practicals
- I have used digital models only because of the restrictions imposed by the COVID-19 pandemic (between March 2020 and March 2021)
- Teaching staff use digital anatomical models for demonstrations
- Other: __________

**Q23. If you have used digital anatomical models previously, what is your opinion on how well these existing resources have supported your learning? If you have not used them before, simply select ‘N/A’.*** [Likert scale]

[A] My institution's own library of digital models (e.g., RVC Learn)

[B] Sketchfab

[C] IVALA Learn, Veterinary Information Network

[D] Virtual Animal Anatomy, Colorado State University

[E] Xerte

All: Great / Good / Neither good nor bad / Bad / Terrible / N/A.

**Q24. Please provide with some reasoning behind your responses to the question above. What makes these digital models useful? What prevents you from using them more?** [optional; free text]

_________________________________________________________

**Q25. Are there any other digital anatomical models / resources we may have missed?** [optional; free text]

_________________________________________________________

## **Section 5: Your ideal digital anatomical model**

This final section is about what features you think the ideal digital model should have to support your learning.

**Q26. Reflecting on your course so far, to what extent do you agree with the following statements?*** [Likert scale]

[A] I would prefer having access to a model with high detail of one specific body part over a model with general details for an entire body

[B] I would prefer having access to models of 3 key species with high levels of anatomical detail over having a larger and more diverse library of species with lower levels of anatomical detail

[C] I would prefer having access to models of an entire juvenile specimen over having a partial model of an adult specimen

All: Strongly disagree / Disagree / Neither agree or disagree / Agree / Strongly agree.

**Q27. Reflecting on what the perfect digital anatomical model would be, pick the top three most important features.*** [option to select multiple up to a maximum of 3]

- The model must have a user interface that is easy to navigate
- The model must have highly detailed annotations (e.g., descriptions of a structure's function)
- The model must be highly detailed (e.g., separate dissections of all anatomical systems)
- The model should meet as many accessibility requirements as possible (e.g., colour-blind friendly colour palette)
- The model must be free to use
- The model must be available offline (i.e., without an internet connection)
- It must be possible to 3D-print the model

All: Strongly disagree / Disagree / Neutral / Agree / Strongly agree.

**Q28. Reflecting on what the perfect digital anatomical model would be, pick the top three most important interactive elements.*** [option to select multiple up to a maximum of 3]

- The model must have the ability to toggle off/on a realistic surface view so that I can test my understanding of where structures begin and end
- The model must have the ability to turn annotations off/on so that I can test my anatomical knowledge
- The model must be available to use on my phone, either as an app or on a website
- The model must be able to be manipulated using a virtual reality headset
- The model must have accompanying animations to demonstrate function (e.g., how the muscles move while the animal is walking/biting).
- The model must have accompanying quiz questions so that I can test my anatomical knowledge

All: Strongly disagree / Disagree / Neutral / Agree / Strongly agree.

**Q29. Are there any other features that you think that the ideal digital anatomical model should have?** [optional; open field]

__________________________________

**Q30. What animal(s) would you benefit from most by having a digital anatomical model?*** [open field]

__________________________________

**Section 6: How well do digital models support your learning?**

This final section asks your opinions on the use digital models would impact your learning during anatomy lectures, practicals, and revision, as well as how digital models could impact wider public understanding of science.

**Q31. Reflecting on your ideal digital anatomical model and your use of existing models so far, to what extent do you agree with the following statements?*** [Likert scale]

[A] Better digital anatomical models are needed in the education of animal anatomy

[B] Being provided with good digital anatomical models while at secondary school / college would have been helpful in preparing for my undergraduate studies

[C] Being provided with good digital anatomical models while at university would better prepare me for my future career

[D] Having digital anatomical models at museum exhibits would improve public understanding of science

[E] Having digital anatomical models of domestic species would enable veterinarians to better communicate with pet owners

All: Strongly disagree / Disagree / Neutral / Agree / Strongly agree.

**Q32. Reflecting on all of your anatomy lectures so far, to what extent do you agree with the following statements?*** [Likert scale]

[A] Using digital models during lectures would be more distracting than helpful

[B] Using digital models during exam revision would be more distracting than helpful

[C] Interacting with digital models would be more useful for revising anatomy than going over written content (lectures, textbooks, notes made during practicals)

[D] Being provided with good digital anatomical models would help me to rely less on my peers

[E] Being provided with good digital anatomical models would help me to rely less on teaching staff

[F] I would be disappointed if ‘digital dissection labs’ replaced some of the anatomy lectures currently scheduled in my curriculum

All: Strongly disagree / Disagree / Neither agree or disagree / Agree / Strongly agree.

**Q33. Reflecting on all of your anatomy practicals so far, to what extent do you agree with the following statements?*** [Likert scale]

[A] Using digital models during practicals would be more distracting than helpful

[B] I wish that the guides I am provided to help with completing the practicals had interactive digital models

[C] Being provided with a digital anatomical model would help me to complete all of the tasks usually set in my anatomy practicals

[D] Being provided with a digital anatomical model would make me feel more confident about working in smaller group sizes during practicals

[F] I would be disappointed if ‘digital dissection labs’ replaced some of the anatomy practicals currently scheduled in my curriculum

All: Strongly disagree / Disagree / Neither agree or disagree / Agree / Strongly agree.

**Q34. Please provide any information that you think would help us to understand your responses to any of the questions above.** [optional; open field]

______________________

## **Section 7: Wrapping up**

Thank you for taking the time to complete this survey. It really is appreciated and – as stated previously – the findings will be valuable for enhancing future students’ experiences.

Here are the final questions. Please remember that the submission of your email address for the future focus groups is entirely optional. This is also the final reminder that this survey is voluntary - your responses will be deleted if you do not click submit upon completion of the survey.

**Q35. Is there anything else you would like to tell us that you have not been able to address in any of your responses above?** [optional; open field]

_____________________________

**Q36. Would you be willing to take part in a focus group to allow us to explore further the issues you have raised in this survey?*** [select one]

Please note that all email addresses submitted for the focus group will be deleted as soon as possible after the meetings have occurred, or as soon as it is decided that focus groups will no longer be going ahead.

- Yes / No

**Q37. Would you like to enter the prize draw to win one of ten £5 vouchers?*** [select one]

All emails submitted for this purpose will be deleted as soon as the prize draw have been completed.

- Yes / No

**Q38. To enable us to contact you regarding the focus groups or prize draw, please enter your University email address.** [optional; open field]

Please check you have typed this accurately before submitting the survey; you do not need to include your email address if you ticked 'No' to the questions above.

____________________________

## **Survey complete**

Thank you for taking the time to complete this survey.

**Appendix B - Staff survey**

The purpose of this study is to investigate how interactive digital models have been used to facilitate the learning of animal anatomy at university level. This online survey is primarily designed for staff who have been involved with teaching at least one in-person anatomy practical to students on veterinary and biosciences courses. We are keen to capture the views of staff in this field from across a diverse community across several universities.

The areas that we are inviting you to respond to are:

- Section 1: About you.
- Section 2: Experience teaching anatomy so far.
- Section 3: Your anatomy practicals.
- Section 4: Your use of anatomical models so far.
- Section 5: What makes the ideal digital anatomical model?
- Section 6: How digital anatomical models impact student learning.

Please read all of the questions and statements carefully.
 **What do you mean by…?** 
When referring to ‘anatomy practicals’, we are primarily discussing the physical dissection, manipulation and/or handling of specimens, whether fresh, fixed (i.e., preserved in formalin), plastinated, skeletonized, fossilized, or as 3D printed replicas. This can include practicals where specimens are handled by only staff, including postgraduate teaching assistants, where students simply observe and take notes. Anatomy practicals can take place in multiple settings (e.g., lab, museum, zoo). 
‘Physical anatomical models’ refer to fixed specimens (Figure 1A) and 3D prints (Figure 1B). In veterinary schools, these are usually found around your building. ‘Digital anatomical models’ refer to interactive 3D structures that can be viewed and manipulated on websites or apps (e.g., SketchFab.com; Figure 1C). Digital models do not include static 2D images on a PowerPoint, for instance. They usually have accompanying annotations and sometimes animations. 

**What's in it for me?** 
It is anticipated that the survey will provide valuable information that will help us to enhance students’ experiences on veterinary and biosciences programmes. There’s also a prize draw to win one of ten £5 vouchers at the end of this survey as a thank you for participating.

**The survey has been kept relatively short (estimated completion time is approximately 15 minutes) so we hope you can find the time to complete it.**

B)

**Figure 1** - Example of the different types of anatomical models commonly used in practicals, demonstrated by the distal limb of a horse. A) a physical anatomical model in the form of a real, skeletonised specimen (image credit: Walter Varcoe); B) a physical anatomical model in the form of a 3D-printed model (image credit: 3D VetAnatomize); C) an interactive digital anatomical model created from a surface scan of a real skeleton (credit: University of Dundee Museum Collections; sketchfab.com).

C)

A)

**Data protection privacy notice** 
The controller for this project is University College London (UCL). The UCL Data Protection Officer provides oversight of UCL activities involving the processing of personal data. If you are concerned about how your personal data is being processed, or if you would like to contact us about your rights, please contact UCL in the first instance at data-protection@ucl.ac.uk 

The information that is required to be provided to participants under data protection legislation (GDPR and DPA 2018) is provided across both the ‘local’ and ‘general’ privacy notices. This ‘local’ privacy notice sets out the information that applies to this particular study. Further information on how UCL uses participant information can be found in our ‘general’ privacy notice https://www.ucl.ac.uk/legal-services/privacy/ucl-general-research-participant-privacy-notice. 

All data will be handled and stored safely according to the General Data Protection Regulations. The lawful basis that will be used to process your personal data, including any special category data is: ‘public task’ and ‘research purposes’. All personal data you provide will be anonymised and we will endeavour to minimise the processing of personal data wherever possible. We will only share findings from this survey in a way that ensures no data will be attributable to an individual, presenting statistical data in the aggregate (collectively). All quotations will be anonymised in any write up. No raw data will be shared with any third parties - only the researcher of this project will have access to datafiles containing potentially identifying information. 

Dr Alice Leavey (Centre for Integrative Anatomy, UCL) is the researcher conducting this survey and performing all data analyses. You can contact her at ucbtal9@ucl.ac.uk if you have any questions about the project. This research has been approved through the central Research Ethics Service at UCL (Ethics ID number 28615/001).

**Q1. I have read and understood the privacy notice.**

Please note: answering 'No' means you will not be able to proceed with the survey.

- Yes / No

**Do I have to provide any personal data?** 
The final section of this survey invites you to opt in to future focus groups which intend to get your feedback on the digital models once they have been made, as well as the opportunity to win a prize for completing the survey. To enter you would need to submit your email address - this is the only identifiable information being collected in this survey and this step is entirely optional. You do not have to provide your email address. Email addresses submitted only for the prize draw will be deleted as soon as the winners are rewarded (approximately one month after the survey deadline). Email addresses submitted for the focus groups will be deleted as soon as these have taken place (approximately three years following this survey depending on the timing of further funding), or as soon as it is decided that the focus groups will no longer go ahead. 

The only special category data you will be asked to provide as part of this survey will be indicating whether you have any additional needs that would need to be considered when creating the digital models (e.g., colour blindness, physical disabilities, neurodivergence). Participants are provided with the option ‘Prefer not to say’, so you do not have to provide this data if you do not wish to.

Consent to participate in this survey will be assumed throughout the completion of the survey. Please note that your participation is completely voluntary, and you can withdraw at any time before submitting the survey. Any incomplete survey responses will be deleted. Once the survey deadline has passed, the anonymous data you provide will be processed only for as long as it is required to complete the wider research project, after which it will then be deleted.

**Q2. I consent to taking part in this survey.**
Please note: answering 'No' means you will not be able to proceed with the survey. By answering ‘yes’, your consent will be assumed throughout the completion of this survey.

- Yes/no

## **Section 1: About you**

This section asks some basic questions about you and your current degree programme. In responding, please remember that – as with the entire survey – your individual responses will be anonymized and we will report on findings in a way that ensures no information will be attributable to any individual.

**Q3. Which best describes you? *** [select one]

If you are a member of staff, do not submit answers to this survey – instead please follow this link to the survey that is designed specifically for you: <https://forms.office.com/e/aWxhAdeHPG>

- Research staff
- Teaching staff
- Anatomy technician
- Other: __________

**Q4. Which year group(s) do you teach animal anatomy to?** Select all that apply. ***** [select one]
Note: Please do not participate in this survey unless you have already taught at least one practical on animal anatomy.

- First
- Second
- Third
- Fourth
- Fifth
- Sixth
- Other: __________

**Q5. Which of the subjects below best represents the degree course you most commonly teach on?*** [select one]

- Biology
- Zoology
- Veterinary Medicine
- Veterinary Nursing
- Bioveterinary
- Other: ___________

**Q6. Do you have experience with anatomy practicals prior to your current role? This can include your time as a student.*** [option to select multiple]

Going forwards, please complete this survey with only your current role involving teaching of anatomy practicals in mind.

- No
- Yes – during school
- Yes – during my first undergraduate degree
- Yes – during my postgraduate degree

**Q7. Please tick all of the following categories that apply to you:*** [option to select multiple]

Please remember that this special category data - as with all the data collected in this survey - will only be reported anonymously and will not be shared with any third parties. Answering this question will be important to enable us to create the most accessible digital models possible.

- I am Neurodivergent (e.g., I have dyslexia, dyspraxia, dyscalculia or ADHD, or am on the autistic spectrum)
- I have a mental health condition (e.g., depression, schizophrenia or anxiety disorder)
- I have a neurological condition (e.g., epilepsy)
- I have a physical impairment or mobility issues, such as difficulty using arms
- I am d/Deaf or have a serious hearing impairment
- I am blind or have a serious visual impairment uncorrected by glasses
- I am colour blind (monochromatism, red-green colour, blue-yellow colour)
- I would prefer not to say
- None of the above
- Other: ____________

**Section 2: Your experiences teaching animal anatomy**

This section is about how you have found learning about the anatomy of animals so far in general. Please remember to read all of the questions and statements carefully.

**Q8. Reflecting on all of your experiences teaching anatomy so far, to what extent do you agree with the following statements?*** [Likert scale]

Select 'N/A' for the final statement if you have not yet taken an assessment on anatomy.

[A] Overall, I find anatomy an enjoyable topic to teach

[B] All body regions are equally difficult to teach in great depth

[C] The anatomy of all animals is equally difficult to teach in great depth

[D] I find it easy to teach how anatomical structures move in 3D space

[E] I find it easy to teach how one small anatomical part fits in with the rest of the larger system

[F] Students often score poorly in assessments on topics related to anatomy

All: Strongly disagree / Disagree / Neutral / Agree / Strongly agree.

**Q9. How difficult are each of these body regions to teach?*** [Likert scale]

If you have taught the body region, select 'N/A'.

[A] Skeletal

[B] Muscular

[C] Digestive

[D] Renal

[E] Nervous

[F] Cardiovascular

[G] Lymphatic

[H] Respiratory

[I] Endocrine

[J] Reproductive

All: Very challenging / Challenging / Neutral / Easy / Very easy / Not applicable.

**Q10. For which animals do you find the anatomy most difficult to teach, and why?*** [free text]

_____________________________

**Q11. Please provide any information that you think would help us to understand your responses to the questions above. In particular, we would love to hear about specific processes, 3D structures, or biomechanical motions that are the most difficult to teach.** [optional; free text]

_____________________________

**Section 3: Your anatomy practicals**

This section includes some basic questions about the structure of your anatomy practicals.

**Q12. On average, how often are students scheduled to have anatomy practicals in an academic year?*** [select one]

- Twice a week
- Weekly
- Monthly
- Once every few months
- Twice a year
- Once a year

**Q13. What are students required to do in advance to prepare for anatomy practicals? Select all that apply.*** [option to select multiple]

- Watch a video demonstration
- Examine a physical anatomical model
- Examine a digital anatomical model
- Review lecture content
- Complete a written assignment
- Deliver a presentation
- Directed reading
- Compare notes with peers
- Complete a quiz
- Nothing
- Other: __________

**Q14. What are students required to do after anatomy practicals to consolidate learning? Select all that apply.*** [option to select multiple]

- Watch a video demonstration
- Examine a physical anatomical model
- Examine a digital anatomical model
- Review lecture content
- Complete a written assignment
- Deliver a presentation
- Directed reading
- Compare notes with peers
- Complete a quiz
- Nothing
- Other: __________

**Q15. On average, how often do students get to do the following activities during practicals?*** [Likert scale]

[A] Physically dissect a fresh/fixed specimen

[B] Physically handle a fresh/fixed specimen

[C] Physically handle a skeleton/fossil

[D] Watch a demonstrator dissect a fresh/fixed specimen

[E] Watch a demonstrator handle a fresh/fixed specimen

[F] Watch a demonstrator handle a skeleton/fossil

[G] Make notes from observing specimens that cannot be handled (e.g., behind a barrier at a museum)

All: Never / Rarely / Sometimes / Often / Always.

**Q16. So far, my anatomy practicals have been…*** [Likert scale]

[A] Engaging

[B] Interactive

[C] Valuable

[D] Safe

[E] Appropriately challenging

[F] Ethical

[G] An important part of the course I teach on

All: Strongly disagree / Disagree / Neutral / Agree / Strongly agree.

**Q17. Are there any other skills, capabilities or awarenesses that you feel the anatomy practicals help students to develop?** [optional; free text]

__________________________________

**Q18. Reflecting on all of your anatomy practicals so far, to what extent do you agree with the following statements?*** [Likert scale]

[A] The current curriculum (e.g., lectures, online learning material) provides sufficient information for students to enter the anatomy practicals feeling prepared

[B] The range of anatomy practicals provided is diverse and interesting

[C] I wish more anatomy practicals were scheduled earlier in the undergraduate course

[D] I wish more anatomy practicals were scheduled later in the undergraduate course

[E] The skills that students learn in anatomy practicals will remain relevant as they go onto the next stage of their career

All: Strongly disagree / Disagree / Neither agree or disagree / Agree / Strongly agree.

**Q19. Reflecting on all your anatomy practicals so far, what aspects do you feel were most beneficial for preparing students for their studies?** [optional; open field]

_____________________________

**Q20. Were there any aspects of your anatomy practicals which you feel could have been improved to better prepare students for their studies?** [optional; open field]

_____________________________

## **Section 4: Your use of digital anatomical models so far**

This section is about the digital models that you have used throughout your teaching to develop your students’ understanding of anatomy.

**Q21. How often do you use physical models, such as fixed specimens or cadavers, to teach anatomy?*** [option to select multiple]

Select all that apply. As a reminder of what a physical anatomical model is, please see Figure 1.

- I have never taught using a physical anatomical model before
- I have never taught using a physical anatomical model before, but will now try to find one
- Students are asked to use physical anatomical models as a part of independent learning (e.g., preparing for lectures or exams)
- Students are asked to use physical anatomical models as a part of my practicals
- I / Other teaching staff use physical anatomical models for demonstrations
- Other: __________

**Q22. How often do you use digital models, such as annotated and interactive 3D models available online, to teach anatomy?*** [option to select multiple]

Select all that apply. As a reminder of what a digital anatomical model is, please see Figure 1C.

- I have never taught using a digital anatomical model before
- I have never taught using a digital anatomical model before, but will now try to find one
- Students are asked to use digital anatomical models as a part of independent learning (e.g., preparing for lectures or exams)
- Students are asked to use digital anatomical models as a part of my practicals
- I have used digital models only because of the restrictions imposed by the COVID-19 pandemic (between March 2020 and March 2021)
- I / Other teaching staff use digital anatomical models for demonstrations
- Other: __________

**Q23. If you have used digital anatomical models previously, what is your opinion on how well these existing resources have supported your teaching? *** [Likert scale]

If you have not used them before, simply select ‘N/A’.

[A] My institution's own library of digital models (e.g., RVC Learn)

[B] Sketchfab

[C] IVALA Learn, Veterinary Information Network

[D] Virtual Animal Anatomy, Colorado State University

[E] Xerte

All: Great / Good / Neither good nor bad / Bad / Terrible / N/A.

**Q24. Please provide with some reasoning behind your responses to the question above. What makes these digital models useful? What prevents you from using them more?** [optional; free text]

_________________________________________________________

**Q25. Are there any other digital anatomical models / resources we may have missed?** [optional; free text]

_________________________________________________________

## **Section 5: Your ideal digital anatomical model**

This final section is about what features you think the ideal digital model should have to support your teaching.

**Q26. Reflecting on your course so far, to what extent do you agree with the following statements?*** [Likert scale]

[A] I would prefer having access to a model with high detail of one specific body part over a model with general details for an entire body

[B] I would prefer having access to models of 3 key species with high levels of anatomical detail over having a larger and more diverse library of species with lower levels of anatomical detail

[C] I would prefer having access to models of an entire juvenile specimen over having a partial model of an adult specimen

All: Strongly disagree / Disagree / Neither agree or disagree / Agree / Strongly agree.

**Q27. Reflecting on what the perfect digital anatomical model would be, pick the top three most important features.*** [option to select multiple up to a maximum of 3]

- The model must have a user interface that is easy to navigate
- The model must have highly detailed annotations (e.g., descriptions of a structure's function)
- The model must be highly detailed (e.g., separate dissections of all anatomical systems)
- The model should meet as many accessibility requirements as possible (e.g., colour-blind friendly colour palette)
- The model must be free to use
- The model must be available offline (i.e., without an internet connection)
- It must be possible to 3D-print the model

All: Strongly disagree / Disagree / Neutral / Agree / Strongly agree.

**Q28. Reflecting on what the perfect digital anatomical model would be, pick the top three most important interactive elements.*** [option to select multiple up to a maximum of 3]

- The model must have the ability to toggle off/on a realistic surface view so that I can test my understanding of where structures begin and end
- The model must have the ability to turn annotations off/on so that I can test my anatomical knowledge
- The model must be available to use on my phone, either as an app or on a website
- The model must be able to be manipulated using a virtual reality headset
- The model must have accompanying animations to demonstrate function (e.g., how the muscles move while the animal is walking/biting).
- The model must have accompanying quiz questions so that I can test my anatomical knowledge

All: Strongly disagree / Disagree / Neutral / Agree / Strongly agree.

**Q29. Are there any other features that you think that the ideal digital anatomical model should have?** [optional; open field]

__________________________________

**Q30. What animal(s) would students benefit from most by having a digital anatomical model?*** [open field]

__________________________________

**Section 6: How well do digital models facilitate student learning?**

This final section asks your opinions on the use digital models would impact your students in terms of their learning during anatomy lectures, practicals, and revision, as well as how digital models could impact wider public understanding of science.

**Q31. Reflecting on your ideal digital anatomical model and your use of existing models so far, to what extent do you agree with the following statements?*** [Likert scale]

[A] Better digital anatomical models are needed in the education of animal anatomy

[B] Providing students with good digital anatomical models while at secondary school / college would have been helpful in preparing them for undergraduate studies

[C] Providing students with good digital anatomical models while at university would better prepare students for their future careers

[D] Having digital anatomical models at museum exhibits would improve public understanding of science

[E] Having digital anatomical models of domestic species would enable veterinarians to better communicate with pet owners

All: Strongly disagree / Disagree / Neutral / Agree / Strongly agree.

**Q32. Reflecting on all of your anatomy lectures so far, to what extent do you agree with the following statements?*** [Likert scale]

[A] Using digital models during lectures would be more distracting for students than helpful

[B] Using digital models during exam revision would be more distracting for students than helpful

[C] Interacting with digital models would be more useful for students revising anatomy than going over written content (lectures, textbooks, notes made during practicals)

[D] Being provided with good digital anatomical models would help students to rely less on their peers

[E] Being provided with good digital anatomical models would help students to rely less on teaching staff

[F] I would be disappointed if ‘digital dissection labs’ replaced some of the anatomy lectures currently scheduled in the students’ curriculum

All: Strongly disagree / Disagree / Neither agree or disagree / Agree / Strongly agree.

**Q33. Reflecting on all of your anatomy practicals so far, to what extent do you agree with the following statements?*** [Likert scale]

[A] Using digital models during practicals would be more distracting for students than helpful

[B] I wish that the guides I provide students to help with completing the practicals had interactive digital models

[C] Providing students with a digital anatomical model would help students to complete all of the tasks usually set in anatomy practicals

[D] Providing students with a digital anatomical model would make me feel more confident about putting students in smaller group sizes during anatomy practicals

[F] I would be disappointed if ‘digital dissection labs’ replaced some of the anatomy practicals currently scheduled in the students’ curriculum

All: Strongly disagree / Disagree / Neither agree or disagree / Agree / Strongly agree.

**Q34. Please provide any information that you think would help us to understand your responses to any of the questions above.** [optional; open field]

______________________

## **Section 7: Wrapping up**

Thank you for taking the time to complete this survey. It really is appreciated and – as stated previously – the findings will be valuable for enhancing future students’ experiences.

Here are the final questions. Please remember that the submission of your email address for the future focus groups is entirely optional. This is also the final reminder that this survey is voluntary - your responses will be deleted if you do not click submit upon completion of the survey.

**Q35. Is there anything else you would like to tell us that you have not been able to address in any of your responses above?** [optional; open field]

_____________________________

**Q36. Would you be willing to take part in a focus group to allow us to explore further the issues you have raised in this survey?*** [select one]

Please note that all email addresses submitted for the focus group will be deleted as soon as possible after the meetings have occurred, or as soon as it is decided that focus groups will no longer be going ahead.

- Yes / No

**Q37. Would you like to enter the prize draw to win one of ten £5 vouchers?*** [select one]

All emails submitted for this purpose will be deleted as soon as the prize draw have been completed.

- Yes / No

**Q38. To enable us to contact you regarding the focus groups or prize draw, please enter your University email address.** [optional; open field]

Please check you have typed this accurately before submitting the survey; you do not need to include your email address if you ticked 'No' to the questions above.

____________________________

## **Survey complete**

Thank you for taking the time to complete this survey.

**Feedback form for the survey pilot test**

| **Instructions** | Please pause at each section of the survey to fill out this form - you can simply put yes or no in each box as indicated, but detailed feedback (including question number) is strongly encouraged. This testing phase is required to meet the requirements for ensuring survey reliability and validity defined by the [*Standards for Educational and Psycological Testing*](https://www.apa.org/science/programs/testing/standards.)*.* Please return this form by Thursday 31st October. Thank you for your feedback! | | | | | | | | | | | | |  |
| --- | --- | --- | --- | --- | --- | --- | --- | --- | --- | --- | --- | --- | --- | --- |
|  | | | | | | | | | | | | | | |
| **Name:** |  | **Institution:** |  | | **Current role:** | |  | | | **Estimated survey completion time:** | |  | |  |
|  | | | | | | | | | | | | | | |
| **Project aims** | Improve our understanding of ...  1. what students are required to do when learning animal anatomy in veterinary and biosciences degrees.  2. how interactive digital resources currently facilitate learning in animal anatomy.  3. the most important criteria for creating the most useful digital anatomical models possible. | | | | | | | | | | | | |  |
|  | | | | | | | | | | | | | | |
| **General questions** | 1. Are the instructions and data protection privacy notice clear and concisely written, such that informed consent to complete the survey can be given? | | | | | | | | | | | Y / N | |  |
|  | 2. Does the order and number of sections make logical sense, such that the survey has a clear flow? | | | | | | | | | | | Y / N | |  |
|  | 3. Is the survey too long? For example, did you feel fatigued by the end of the survey? | | | | | | | | | | | Y / N | |  |
|  | 4. Does the survey accurately cover all aspects of the concepts we are trying to measure (i.e., importance of digital anatomical models, where digital anatomical models require improvement)? | | | | | | | | | | | Y / N | |  |
|  | | | | | | | | | | | | | | |
| **Section-specific questions** | | **Section 1: About you** | | **Section 2: Experience learning anatomy** | | **Section 3: How anatomy practicals support learning** | | **Section 4: Anatomy assessments and exams** | **Section 5: Your use of digital anatomical models** | | **Section 6: What makes the ideal digital anatomical model?** | | **Section 7: Wrapping up** | |
| 1. Are the statements clearly and concisely written, such that questions are straightforward to answer? There should be no spelling mistakes, double-barrelled questions, double-negative questions, double-meanings, or overly long questions. | | Y / N | | Y / N | | Y / N | | Y / N | Y / N | | Y / N | | Y / N | |
| 2. Are there any questions you believe to be ‘leading' or biased? | | Y / N | | Y / N | | Y / N | | Y / N | Y / N | | Y / N | | Y / N | |
| 3. Is there any unexplained jargon or language that is difficult to follow? | | Y / N | | Y / N | | Y / N | | Y / N | Y / N | | Y / N | | Y / N | |
| 4. Have all the questions been assigned the most appropriate format (i.e., multiple choice, Likert scale, ranking, open-text)? | | Y / N | | Y / N | | Y / N | | Y / N | Y / N | | Y / N | | Y / N | |
| 5. Are all the questions essential and appropriate to fully address the survey project aims? | | Y / N | | Y / N | | Y / N | | Y / N | Y / N | | Y / N | | Y / N | |
|  | | | | | | | | | | | | | | |
| **Please write any additional feedback here:** |  | | | | | | | | | | | | |  |

**Supplementary tables**

**Table S1** – Results of the Cronbach’s alpha test of reliability for each theme in the survey, where negatively worded statements have been reverse-coded to ensure the same directionality between statements. ‘Challenge data’ refers to where participants were asked to rate how challenging each area of the body is to teach / learn. Several areas were non-applicable to many participants, so we also analysed Cronbachs alpha for the theme of general experiences with anatomy with and without these data.

| **Survey themes** | **Cronbach's alpha** | **Notes** |
| --- | --- | --- |
| General experiences with anatomy | 0.434 | No challenge data = 0.514; challenge data only = 0.731 |
| Practicals - information | 0.77 |  |
| Practicals - opinions | 0.804 |  |
| Ideal digital anatomical model | 0.363 | Low question number (n = 3) |
| Impacts of digital anatomical models | 0.761 |  |

**Table S2** – The result of the chi-squared tests that showed significant differences in the answers to multiple choice questions between predictor variables. The staff survey responses showed no significant differences.

| **Dataset** | **Question** | **Independent variable** | **Chi-squared** | ***p*-value** | **Top three contributors (residual)** |
| --- | --- | --- | --- | --- | --- |
| **Student survey responses** | What are you required to do after anatomy practicals to consolidate learning? | Participant type | 61.36 | 0.001 | Presentation x Postgraduate student (5.83);  Written assignment x Recent graduate (3.05);  Nothing x Undergraduate (second degree) (2.93) |
|  | What are you required to do after anatomy practicals to consolidate learning? | Year group | 60.23 | 0.007 | Presentation x Fourth (5.83);  Written assignment x Graduate (3.05);  Directed reading x Graduate (2.06) |
|  | How often do you use physical models, such as skeletons or fixed specimens, to learn anatomy? | Subject | 22.67 | 0.007 | Not used x Biology (3.76);  Not used x Veterinary Medicine (-3.52);  Independent learning x Veterinary Medicine (2.2) |
|  | How often do you use digital models, such as annotated and interactive 3D models available online, to learn anatomy? | Subject | 26.47 | 0.033 | Not used x Veterinary Medicine (-3.58);  Not used x Biology (3.47);  Not used x Zoology (2) |
|  | Reflecting on what the perfect digital anatomical model would be, pick the top three most important features. | Subject | 29.52 | 0.042 | Accessibility features x Veterinary Nursing (3.98);  Available offline x Veterinary Medicine (2.29);  Easy to navigate x Biology (1.98) |
| **Comparing student and staff responses** | What are you required to do in advance to prepare for anatomy practicals? | Group | 19.89 | 0.019 | Compare notes x staff (2.75);  Compare notes x student (-2.75);  Other x staff (1.95) |
|  | Reflecting on what the perfect digital anatomical model would be, pick the top three most important features. | Group | 15.10 | 0.018 | Accessibility features x staff (2.43);  Accessibility features x student (-2.43);  3D printable x staff (2.16) |

**Supplementary Figures**

**Figure S1 –** The results of Kendall’s Tau-b analysis, which compares the strength of association between each pair of Likert scale questions. Blue tones represent a significant positive relationship, red tones indicate a significant negative relationship, and blank boxes indicate no significant relationship. The question asking participants to rate how useful the digital anatomical models are on SketchFab had to be removed due to the presence of too many NAs. Note that the questions have been shortened/amended to fit within the plot space – full versions of each question can be found in Appendices A and B.

Engaging

Interactive

Valuable

Safe

Appropriately challenging

An important part of the degree course

**So far, my anatomical practicals have been…**

Ethical

*

**Figure S2** – Participant views about how they find specific aspects of their current anatomy practicals. The black asterisk represents a significant difference in how students interpreted the safety of their anatomy practicals. First-year students agreed more strongly that their anatomy practicals are safe than second-year students (p<0.05; Table S2).

|  | Count |
| --- | --- |
| Exotics | 6 |
| Humans | 5 |
| Equine | 4 |
| Fish | 3 |
| Large animals | 2 |
| Non-mammals | 2 |
| Reptiles | 2 |
| Cattle | 2 |
| Musculoskeletal | 2 |
| Ruminant gut | 2 |
| All | 1 |
| Vertebrates | 1 |
| Invertebrates | 1 |
| Rare animals | 1 |
| Livestock | 1 |
| Birds | 1 |
| Mammals | 1 |
| Small mammals | 1 |
| Marine animals | 1 |
| Microscopic organisms | 1 |
| Rabbits | 1 |
| Nematodes | 1 |
| Flies | 1 |
| Squid | 1 |
| Annelids | 1 |
| Turtle | 1 |
| Nervous system | 1 |
| Lungs | 1 |
| Reptile - respiration | 1 |
| Reptile - reproduction | 1 |
| Anatomical planes | 1 |
| Descriptions of movement | 1 |

**Figure S3** – A summary of the number of times each response was mentioned for the open-text question: *For which animals do you find the anatomy most difficult to learn / teach?* The responses are colour-coded by the categories ‘general’ (yellow), ‘large animal group’ (green), ‘specific animal’ (orange), ‘anatomical system’ (red), and ‘biomechanical’ (grey).

My institution’s own library of digital models

(n = 18, NA = 25)

IVALA Learn, Veterinary Information Network

(n = 9, NA = 34)

SketchFab

(n = 7, NA = 36)

Virtual Animal Anatomy, Colorado State University

(n = 9, NA = 34)

Xerte

(n = 3, NA = 40)

**Figure S4** – Likert scale ratings of existing digital anatomical models. These data could not be statistically analysed due to low sample size (total number of participants = 43), as many staff and students had not previously used or were not aware of these resources.

|  | Count |
| --- | --- |
| Dog | 13 |
| Humans | 11 |
| Horse | 9 |
| Cat | 9 |
| Fish | 6 |
| All/variety | 4 |
| Cow | 4 |
| Birds | 3 |
| Cephalopod/squid | 3 |
| Domestic animals | 2 |
| Large animals | 2 |
| Farm animals/Livestock | 2 |
| Reptiles | 2 |
| Fly | 2 |
| Sheep | 2 |
| Chicken/Poultry | 2 |
| Dolphin | 2 |
| Pregnant animal | 1 |
| Invertebrates | 1 |
| Mammals | 1 |
| Exotics | 1 |
| Amphibians | 1 |
| Primates | 1 |
| Pig | 1 |
| Mouse | 1 |
| Rabbit | 1 |
| Guinea pig | 1 |
| Rat | 1 |
| Shark | 1 |
| Ferret | 1 |
| Chinchilla | 1 |
| Locust | 1 |
| Sea mouse | 1 |
| Wasp | 1 |
| Pterosaur | 1 |
| Crocodile | 1 |
| Bat | 1 |
| Ichthyosaur | 1 |
| Penguin | 1 |
| Platypus | 1 |

**Figure S5** – A summary of the number of times each response was mentioned for the open-text question: *Which animal(s) would students benefit from most by having a digital anatomical model?* The responses are colour-coded by the categories ‘general’ (yellow), ‘large animal group’ (green), and ‘specific animal’ (orange).
